# Pleiotropic Odorant-Binding Proteins Promote Aedes aegypti Reproduction and Flavivirus Transmission

**DOI:** 10.1128/mBio.02531-21

**Published:** 2021-10-12

**Authors:** Shengzhang Dong, Zi Ye, Chinmay Vijay Tikhe, Zhijian Jake Tu, Laurence J. Zwiebel, George Dimopoulos

**Affiliations:** a W. Harry Feinstone Department of Molecular Microbiology and Immunology, Bloomberg School of Public Health, Johns Hopkins University, Baltimore, Maryland, USA; b Department of Biological Sciences, Vanderbilt University, Nashville, Tennessee, USA; c Department of Biochemistry, Virginia Tech, Blacksburg, Virginia, USA; National Institute of Allergy and Infectious Diseases

**Keywords:** *Aedes aegypti*, Zika virus, dengue virus, odorant-binding proteins, OBP10, OBP22, blood feeding, feeding behavior, reproduction, virus transmission

## Abstract

Insect odorant-binding proteins (OBPs) are small soluble proteins that have been assigned roles in olfaction, but their other potential functions have not been extensively explored. Using CRISPR/Cas9-mediated disruption of Aedes aegypti *Obp10* and *Obp22*, we demonstrate the pleiotropic contribution of these proteins to multiple processes that are essential for vectorial capacity. Mutant mosquitoes have impaired host-seeking and oviposition behavior, reproduction, and arbovirus transmission. Here, we show that *Obp22* is linked to the male-determining sex locus (M) on chromosome 1 and is involved in male reproduction, likely by mediating the development of spermatozoa. Although OBP10 and OBP22 are not involved in flavivirus replication, abolition of these proteins significantly reduces transmission of dengue and Zika viruses through a mechanism affecting secretion of viral particles into the saliva. These results extend our current understanding of the role of insect OBPs in insect reproduction and transmission of human pathogens, making them essential determinants of vectorial capacity.

## INTRODUCTION

Aedes aegypti is the major vector for many infectious diseases caused by arthropod-borne viruses (arboviruses) such as dengue virus (DENV), Zika virus (ZIKV), chikungunya virus (CHIKV), and yellow fever virus (YFV) (1). These pathogens cause hundreds of millions of global infections yearly that result in a tremendous health and socioeconomic burden (2, 3). The lack of effective vaccines and antiviral medications for key arboviral diseases renders the development of novel disease control approaches urgent. Toward this goal, a better understanding of the biology of mosquitoes and their interactions with their pathogens is clearly desirable (4–8).

Completion of the arbovirus transmission cycle requires blood feeding by female mosquitoes, as well as successful interactions between the pathogen and various mosquito organs. Female A. aegypti mosquitoes use their chemosensory systems, along with other sensory modalities, to locate their vertebrate blood meal host and find a microcapillary blood vessel, allowing them to acquire the blood that is necessary for egg production (9). During this process, mosquitoes are able to transfer viruses to/from the host and thereby enable pathogen transmission (10). Numerous studies have demonstrated that the mosquito’s olfactory and gustatory systems play a crucial role in host seeking, blood feeding, and oviposition behaviors, which involve a large array of signal transduction and neuronal processing components (11–14). Of these, odorant-binding proteins (OBPs), which often comprise the most prominently expressed proteins present in the chemosensory hairs (sensilla) where signal transduction occurs, remain largely enigmatic. Insect OBPs are small (<30-kDa) soluble proteins that are secreted by sensillar accessory cells into the aqueous lymph surrounding the olfactory sensory neurons (OSNs) of the antennae, maxillary palps, proboscis, and other sensory appendages, where they are likely to be the first contact between semiochemicals and the olfactory sensory system (11, 15). The major function of insect OBPs is believed to be the solubilization and chaperoning of external odorants, followed by the transport of these odorants to OSN membrane-bound odorant receptors (ORs), where they can initiate peripheral signal transduction (16).

Mosquito genomes encode a large number of *Obp* genes (17), and the first mosquito OBP was isolated from the antennae of a female Culex quinquefasciatus mosquito (18). At least 111 putative *Obp* genes have been identified in the A. aegypti genome (17, 19), and these *Obp* genes are expressed in multiple tissues (20), suggesting that the OBPs have other physiological functions beyond the olfactory system. This hypothesis was supported by recent studies of the three-dimensional structure of several mosquito OBPs that yielded data suggesting that these OBPs can bind to a diversity of small molecules (21–24). However, the biological functions of these molecules beyond their hypothesized roles in olfaction have not yet been demonstrated in any mosquito species (11).

We have previously shown that the abundance of transcripts encoded by the A. aegypti *Obp* genes *Obp10* and *Obp22* are modulated by dengue virus serotype 2 (DENV2) infection, and their RNA interference (RNAi)-mediated knockdown significantly and adversely affects the host-seeking and blood-feeding behaviors of female mosquitoes (25). In addition, OBP22 is able to bind to a broad range of fatty acids and other small-molecule ligands (26, 27), suggesting that it is likely to have multiple functions in mosquitoes. Here, we used CRISPR/Cas9-based genome editing to functionally delete (knock out) *Obp10* and *Obp22* in A. aegypti to investigate their respective involvement in blood feeding, oviposition, reproduction, development, and arbovirus transmission. Our studies reveal *Obp10* and *Obp22* play diverse pleiotropic roles that are essential for maintaining the vectorial capacity of A. aegypti.

## RESULTS

### Generation and characterization of *Obp10* or *Obp22* knockout mutants.

**FIG 1:**
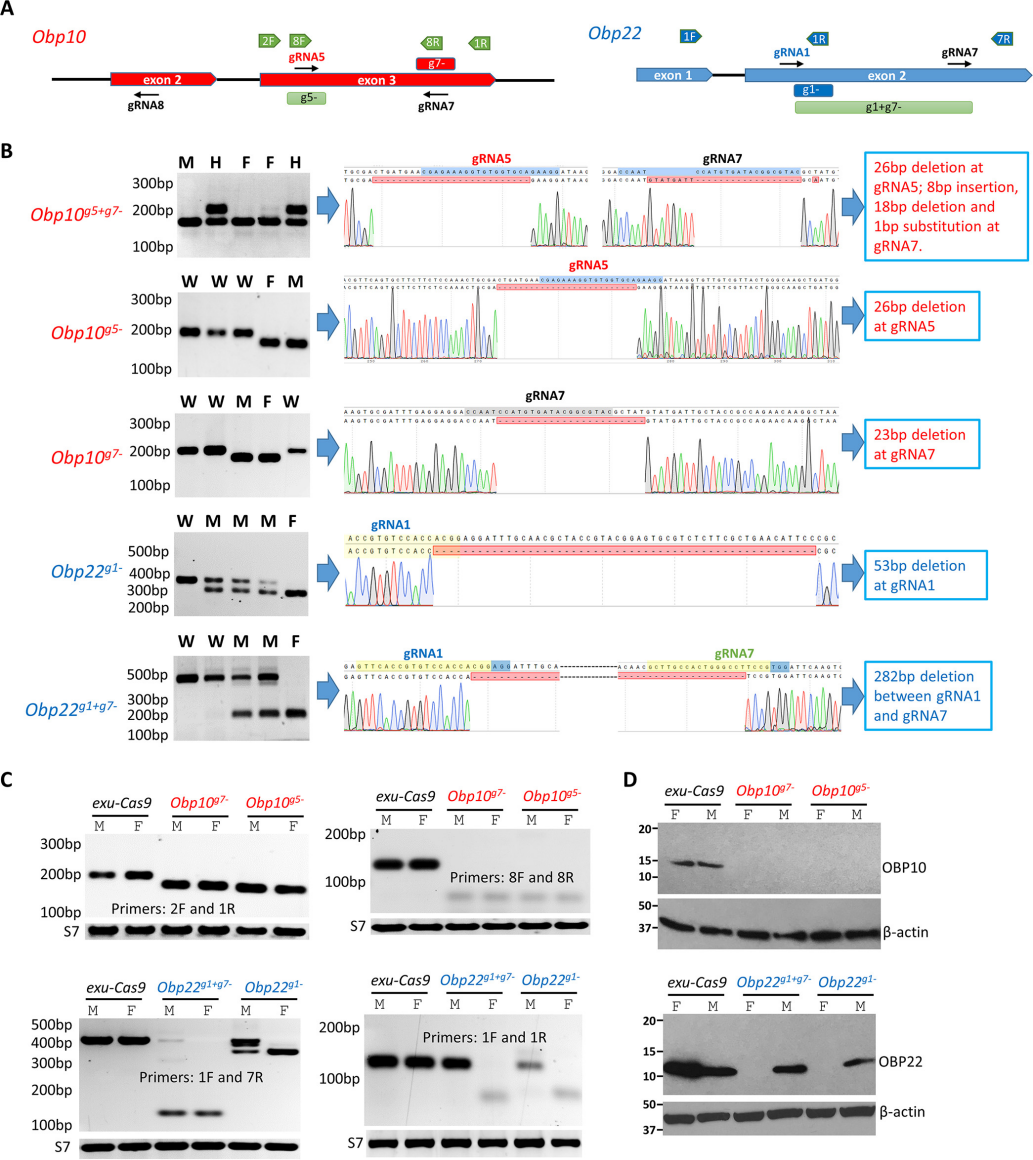
CRISPR/Cas9-mediated knockout (KO) of *Obp10* or *Obp22* in Aedes aegypti. (A) Schematic representation of gRNA targets and mutation of *Obp10* and *Obp22* and primers used for reverse transcription-PCR (RT-PCR). (B) Amplification of *Obp* in mutants (left), sequencing trace data for the *Obp10* or *Obp22* mutation (middle), and deletions or insertions of *Obp* mutants (right). In comparison to the wild-type (WT) band, a truncated band was considered to be a mutation, which was then confirmed by subsequent Sanger sequencing. W, wild type; H, heterozygous mutants; M, mutated males; F, mutated females. (C) RT-PCR amplification of *Obp* transcripts in mutants with different primer sets; *S7* was used as a PCR amplification control. (D) Western blot detection of OBP10 (upper) and OBP22 (lower) protein in *Obp* KO and *exu-Cas9* females and males with their corresponding polyclonal antibodies. β-Actin was used as a protein loading control.

Three guide RNAs (gRNA5, gRNA7, and gRNA8) targeting the A. aegypti *Obp10* gene (Fig. 1A) were individually injected into eggs of the *Cas9* mosquitoes (*exu-Cas9*), and mutation efficiency in G0 survivals were determined using a mosquito leg PCR and Sanger sequencing (see Table S1 in the supplemental material). Subsequently, we injected both gRNA5 and gRNA7 into the eggs to obtain a large deletion between these two gRNAs. The mutated G0 female survivors were crossed with *exu-Cas9* males to maintain the same genetic background as the *exu-Cas9* line. After outcrossing of the *Obp10* heterozygous mutants with *exu-Cas9* mosquitoes for another three generations, we incrossed the heterozygous mutants to obtain *Obp10^−/−^* homozygotes. Using this method, we established at least three genotypes of *Obp10* knockout (KO) homozygotes. Sequencing data showed that gRNA5 and gRNA7 induced genotypes/mutations individually (*Obp10^g5−^* by gRNA5 and *Obp10^g7−^* by gRNA7) or concurrently (*Obp10^g5+g7−^*) (Fig. 1B).

10.1128/mBio.02531-21.8 TABLE S1 Data from generating *Obp10* or *Obp22* knockout mosquitoes. Guide RNAs (gRNAs) targeting *Obp10* or *Obp22* were microinjected into eggs of the *exu-Cas9* females. The hatching rate was calculated by dividing the number of hatched eggs by the total number of eggs injected for each gRNA or gRNA mix. Mutation of the injected adults (G0) was determined by a leg PCR and/or Sanger sequencing. Mutation rate was calculated by dividing the number of mutated G0 mosquitoes by the total number of G0 mosquitoes surviving for each gRNA or gRNA mix. Download Table S1, PDF file, 0.5 MB. Copyright © 2021 Dong et al. 2021 Dong et al. https://creativecommons.org/licenses/by/4.0/ This content is distributed under the terms of the Creative Commons Attribution 4.0 International license.

Two gRNAs (gRNA1 and gRNA7) were designed to target *Obp22* (Fig. 1A). Using the same crossing and screening strategy that we followed for the *Obp10* KO, we generated two female genotypes of *Obp22* KO homozygotes, *Obp22^g1−^*, with a 53-bp deletion induced by gRNA1, and *Obp22^g1+g−^*, with a 282-bp deletion induced concurrently by both gRNA1 and gRNA7 (Fig. 1B). It is noteworthy that the *Obp22* KO males have a heterozygous genotype (one KO allele and one wild-type [WT] allele); females are homozygous (two KO alleles) (Fig. 1B), indicating that *Obp22* may be linked to the male-determining sex locus (M) on chromosome 1.

To determine whether the mutations described above affect the transcription and function of the *Obp10* and *Obp22* genes, we performed reverse transcription-PCR (RT-PCR) to amplify the *Obp10* and *Obp22* transcripts in *Obp* KO mosquitoes. Truncated transcripts were amplified in *Obp10* KO and *Obp22* KO homozygotes (Fig. 1C), showing a reading-frame shift or a stop codon in the middle of *Obp10* or *Obp22*, and these mutations affected the predicted conserved domain of *Obp* (Fig. S1). As a stop codon halted protein translation, OBP10 or OBP22 protein expression was completely depleted in *Obp10* or *Obp22* KO mosquitoes, respectively, except in the heterozygous *Obp22* KO males, which had a reduced expression of the OBP22 protein compared to that in *exu-Cas9* males, as determined by Western blotting with specific antibodies against OBP10 or OBP22 in A. aegypti (Fig. 1D).

10.1128/mBio.02531-21.1 FIG S1 Reading frames of truncated *Obp* transcripts in *Obp10* or *Obp22* knockout mosquitoes. (A) Predicted functional domain (upper) and reading frame of wild-type *Obp10* (middle), and truncated *Obp10* reading frames of *Obp10^g7−^* and *Obp10^g5−^* homozygotes (lower). (B) Predicted functional domain (upper) and reading frame of wild-type *Obp22* (middle) and truncated *Obp22* reading frames of *Obp22^g1−^* and *Obp22^g1+g7−^* homozygotes (lower). Download FIG S1, PDF file, 0.6 MB. Copyright © 2021 Dong et al. 2021 Dong et al. https://creativecommons.org/licenses/by/4.0/ This content is distributed under the terms of the Creative Commons Attribution 4.0 International license.

Thus, using CRISPR/Cas9, we successfully mutated *Obp10* and *Obp22* in A. aegypti and completely depleted OBP10 function in both females and males, as well as completely depleting OBP22 function in females and partially depleting OBP22 function in males.

### Disruption of *Obp10* or *Obp22* does not affect female mosquito antennal responses to key odorants.

**FIG 2:**
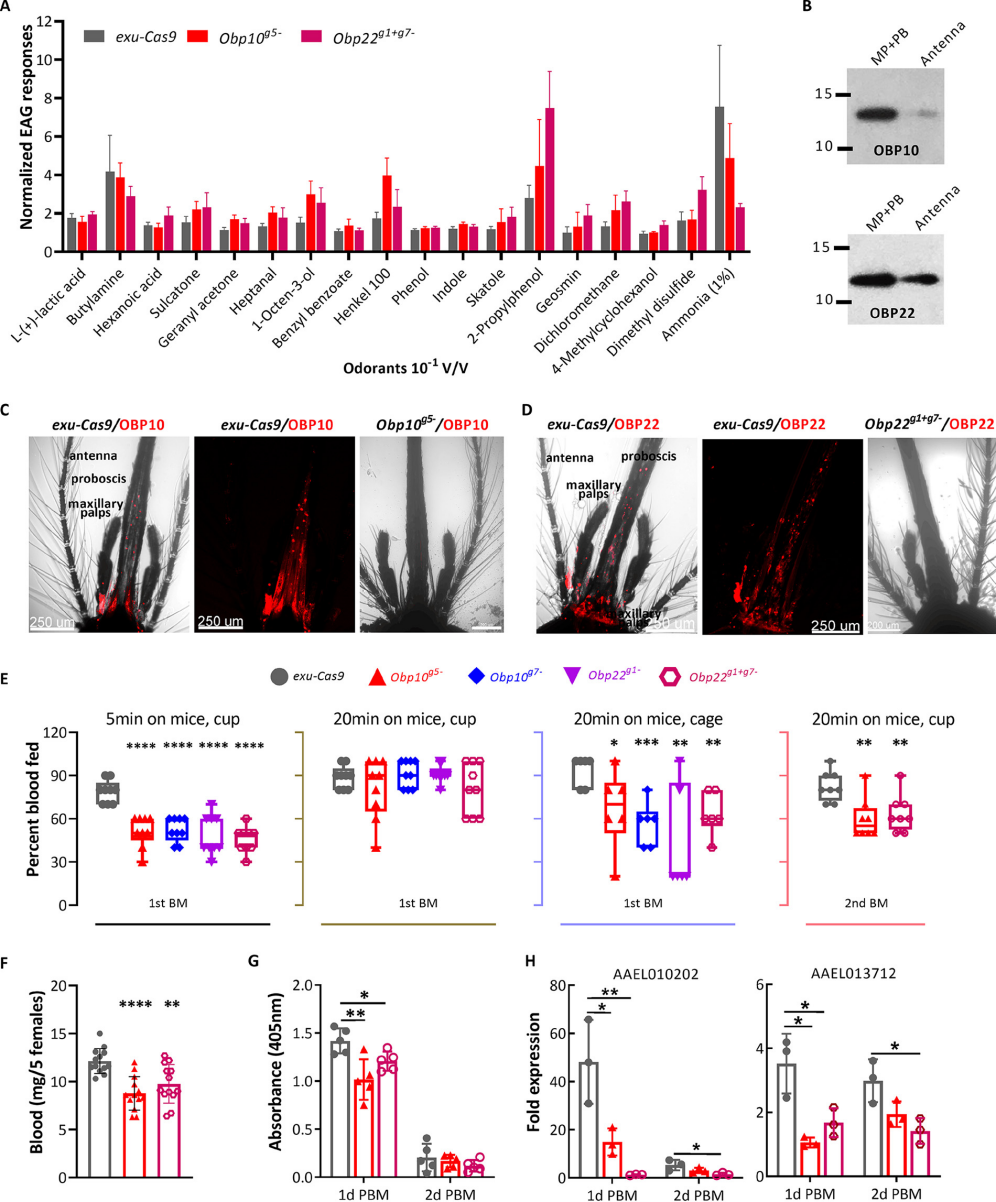
Knockout of *Obp10* or *Obp22* reduces blood-feeding capability but does not affect the electroantennogram (EAG) responses of female A. aegypti mosquitoes. (A) EAG responses to the selected odorants. (B) Western blot detection of OBP10 and OBP22 in mouthparts (maxillary palps [MP] and proboscis [PB]) and antenna of *exu-Cas9* females. Each sample includes 20 antennae or mouthparts collected from 4- to 5-day-old females. (C and D) Immunofluorescence assay (IFA) detection of OBP10 and OBP22 in the antennae and mouthparts of *exu-Cas9* and *Obp* KO females with their corresponding antibody (red). (E) Percentage of females that were fed on mice in a cup for 5 min or 20 min or in a cage for 20 min, or those who received the second blood meal (BM) in a cage for 20 min. (F) Amount of blood taken by five females in a cage for 20 min. Three technical repeats with three biological replicates were performed. Data from different biological replicates were pooled and used for generating dotted graphs. (G) Endogenous trypsin activity in midguts of females at 1 and 2 days post blood meal (PBM). (H) Reverse transcription-quantitative PCR (qRT-PCR) detection of trypsin gene expression in females at 1 and 2 days PBM.

To first determine whether OBP10 or OBP22 is broadly implicated in olfactory signal transduction, we used electroantennogram (EAG) analyses to perform a comparative survey of responses to a panel of 18 volatile odorants across the antennae, which are the principal olfactory appendages on adult A. aegypti mosquitoes. These studies did not reveal significant differences in EAG responses to any of the selected odorants between *Obp10^g5−^* or *Obp22^g1+g7−^* and *exu-Cas9* (parental WT control) females (Fig. 2A), indicating that OBP10 and OBP22 are not involved in antennal olfactory processes. We next examined the expression of OBP10 and OBP22 on the antennae, as well as that on the maxillary palps and proboscis mouthparts, which act as accessory chemosensory appendages for adult females, by Western blotting and immunofluorescence assay (IFA). While low levels of OBP10 and modest levels of OBP22 were detected in extracts of *exu-Cas9* female antennae, strong signals for both were obtained with mouth part protein extracts (Fig. 2B). IFA results confirmed that OBP10 and OBP22 were weakly expressed in the antennae (Fig. S2) but highly expressed in the mouthparts, particularly in the proboscis of *exu-Cas9* females but not in those of *Obp10^g5−^* or *Obp22^g1+g7−^* females (Fig. 2C and D), and that the antennae are not the major chemosensory organs expressing OBP10 or OBP22.

10.1128/mBio.02531-21.2 FIG S2 Immunofluorescence assay (IFA) detection of OBP10 (A) and OBP22 (B) in the antennae of *exu-Cas9* females with their corresponding antibody (red). Zoomed areas are indicated by white boxes. Download FIG S2, PDF file, 0.5 MB. Copyright © 2021 Dong et al. 2021 Dong et al. https://creativecommons.org/licenses/by/4.0/ This content is distributed under the terms of the Creative Commons Attribution 4.0 International license.

### Disruption of *Obp10* or *Obp22* impairs blood feeding.

To investigate a potential role for *Obp10* or *Obp22* in blood feeding, we fed the *Obp* KO homozygotes, along with WT control (*exu-Cas9*) mosquitoes, on mice for 5 min or 20 min in a 16- oz cup or in a larger (length = 8 in., width = 8 in., height = 8 in.) cage. In cup assays with five females exposed to mice for 5 min, only 51.1% of *Obp10^g7−^*, 52.2% of *Obp10^g5−^*, 48.9% of *Obp22^g1−^*, and 45.6% of *Obp22^g1+g7−^* homozygotes fed, whereas 78.9% of the control females fed (Fig. 2E). When the *Obp* KO mosquitoes were exposed to a mouse for a prolonged time (20 min) in the cup bioassay, there was no significant difference in the percentage of blood-fed mosquitoes between either of the *Obp* KO mosquitoes and control mosquitoes. However, when five females were allowed a prolonged (20 min) feeding in cage assays, the percentages of blood-fed *Obp10* (66.7% of *Obp10^g7−^* and 56.7% of *Obp10^g−^*) or *Obp22* (43.3% of *Obp22^g1−^* and 63.3% of *Obp22^g1+g7−^* KO homozygotes were significantly lower than that of the control mosquitoes (93.3%) (Fig. 2E). When the mosquitoes were provided with a second blood meal 5 days after the first blood feeding and subsequent egg production and oviposition, the percentage of blood-fed *Obp10* or *Obp22* KO homozygotes was slightly higher but was still significantly lower than that of the control females (Fig. 2E).

We also assessed the effect of the *Obp* mutations on the amount of ingested blood from groups of 5 individuals who successfully blood fed in these bioassays. Here, *Obp10^g5−^* and *Obp22^g1+g7−^* mosquitoes acquired approximately 8.8 μg and 9.8 μg blood, respectively, which in both instances is significantly less than *exu-Cas9* control mosquitoes, which ingested approximately 12.1 μg blood (Fig. 2F), indicating the amount of ingested blood was significantly reduced in *Obp* KO mosquitoes. To also determine the effect of *Obp10* or *Obp22* disruption on blood digestion, we measured the midgut trypsin activity in the *Obp* KO and control mosquitoes at 1 and 2 days post blood meal (PBM). The results showed that the overall midgut trypsin activity in the *Obp10^g5−^* and *Obp22^g1+g7−^* mosquitoes was significantly lower than that in control females at 1 day PBM (Fig. 2G) and that at 2 days PBM, trypsin activity levels were dramatically reduced for all genotypes. Consistent with this decreased trypsin activity, the expression of several trypsin genes, including AAEL010202 and AAEL013712, was also significantly reduced in the *Obp10* and *Obp22* KO mosquitoes at 1 day PBM (Fig. 2H). It is likely that the activity of additional proteases is also affected by *Obp* disruption. Taken together, these data indicate that capacity to acquire and digest a blood meal is significantly impaired in the *Obp10* and *Obp22* KO mutants.

### Disruption of *Obp10* or *Obp22* impairs mosquito reproduction.

**FIG 3:**
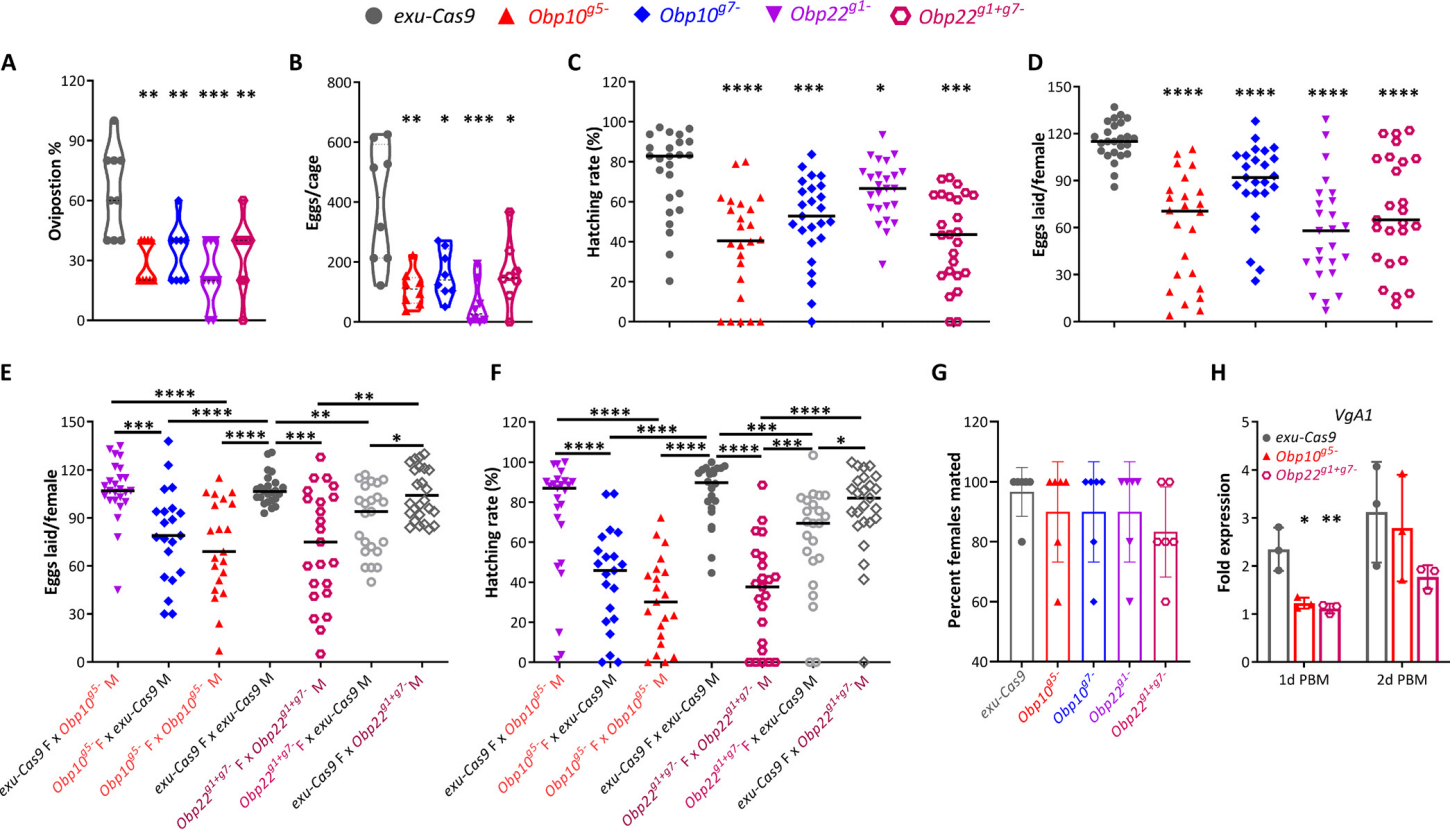
Female fecundity and fertility are reduced in *Obp10* or *Obp22* knockout females. (A) The percentage of oviposition was determined by calculating the ratio of oviposited females to all of the females in a cage. (B) Number of eggs laid per five females in a cage overnight. (C and D) Numbers of eggs and their hatching rates in *Obp* KO and *exu-Cas9* females. (E and F) Numbers of eggs and their hatching rates in *Obp* KO and *exu-Cas9* females (F) that were mated with *Obp* KO or *exu-Cas9* males (M). (G) Percentage of mated females. Five pairs of males or females were mated in a cage overnight, and their mating success was determined by monitoring the offspring hatching or by amplifying *Nix* in female spermathecae. (H) qRT-PCR detection of *VgA1* expression in females at 1 or 2 days post blood meal (PBM). At least two replicates were performed for each graph. *P* values for *Obp* KO lines versus the control *exu-Cas9* line were determined by using an unpaired *t* test (A, B, G, and H) or a Mann-Whitney test (C to F) with GraphPad Prism. *, *P < *0.05; **, *P < *0.01; ***, *P < *0.001; ****, *P < *0.0001.

**(i) Impairment of oviposition.** To determine whether disruption of *Obp10 or Obp22* affects oviposition in A. aegypti, we measured the proportion of females laying eggs at 3 days PBM. In these assays, five females of a given genotype were introduced into a cage (length = 8 in., width = 8 in., height = 8 in.) with an oviposition cup containing filter paper on top of distilled water (dH_2_O). After 24 h, the number of oviposited females was counted, as well as the number of eggs laid on the filter paper. In these assays, all genotypes of *Obp* KO mosquitoes showed a significantly lower oviposition percentage than did the *exu-Cas9* control mosquitoes (Fig. 3A). In particular, the oviposition rates of *Obp10^g5−^* and *Obp22^g1+g7−^* mosquitoes were 27.5% and 20%, respectively, whereas that for the *exu-Cas9* mosquitoes was 62.5%. Consistent with the reduced oviposition frequency, the numbers of eggs deposited on filter paper substrates across all of the *Obp* KO mutant mosquitoes were also significantly lower than those for controls (Fig. 3B).

**(ii) Reduction in the fecundity and fertility of female mosquitoes.** In addition to several other considerations, mosquito fecundity is dependent on the amount of blood ingested. Since blood feeding is impaired in *Obp* KO mutants, we investigated the possible impact of the mutations on fecundity. Compared to the control (*exu-Cas9*) females, *Obp10* or *Obp22* KO females not only oviposited a significantly lower number of eggs, but the hatching rate of those laid was also significantly reduced (Fig. 3C and D). To determine whether the reduced fecundity and fertility were the result of a gender-specific impairments in either the *Obp* KO females or males, we mated the *Obp* KO females with *exu-Cas9* males and *Obp* KO males with *exu-Cas9* females and subsequently assessed the numbers of laid and hatched eggs. Compared to the *exu-Cas9* females, *Obp10^g5−^* and *Obp22^g1+g7−^* females produced significantly lower numbers of eggs (Fig. 3E), with significantly lower hatching rates (Fig. 3F), when they were crossed with same-genotype *Obp* KO males or *exu-Cas9* males. In addition, the hatching rates of eggs produced by *Obp22^g1+g7−^* females that were crossed with *Obp22^g1+g7−^* males were significantly lower than those of the same strain of females crossed with *exu-Cas9* males, suggesting that the *Obp22* mutation also affects the fertility of males (Fig. 3F). These data suggest that the impaired fecundity and fertility of *Obp10* KO mosquitoes can be attributed to defects in the females, but the reduced fecundity and fertility in *Obp22* KO mosquitoes can likely be attributed to both females and males.

To determine whether the reduced fecundity and fertility in *Obp* KO mosquitoes is related to the biology of male mating, we assessed the mating success of *Obp* KO males by measuring the offspring hatch rate and/or by amplifying the male-specific gene *Nix* in female spermathecae as an indirect measure of sperm present in that organ. By these measures in a cage (length = 8 in., width = 8 in., height = 8 in.) with five pairs of females and males left for overnight mating, the percentage of *Obp* KO females that had mated was not significantly lower than that for the control *exu-Cas9* females (Fig. 3G), suggesting that neither *Obp10* nor *Obp22* influences male mating success.

To further assess influence on reproduction, we also assayed the expression of the *VgA1* gene, which encodes the major egg yolk protein vitellogenin, in *Obp* KO and *exu-Cas9* female mosquitoes by quantitative PCR (qPCR) and found that *VgA1* expression was significantly decreased in *Obp10* and *Obp22* KO females compared to that in control females (Fig. 3H).

In summary, *Obp10* or *Obp22* significantly influenced female fecundity and fertility, but did not affect male mating success.

### OBP22 affects male reproduction by regulating the development of spermatozoa.

**FIG 4:**
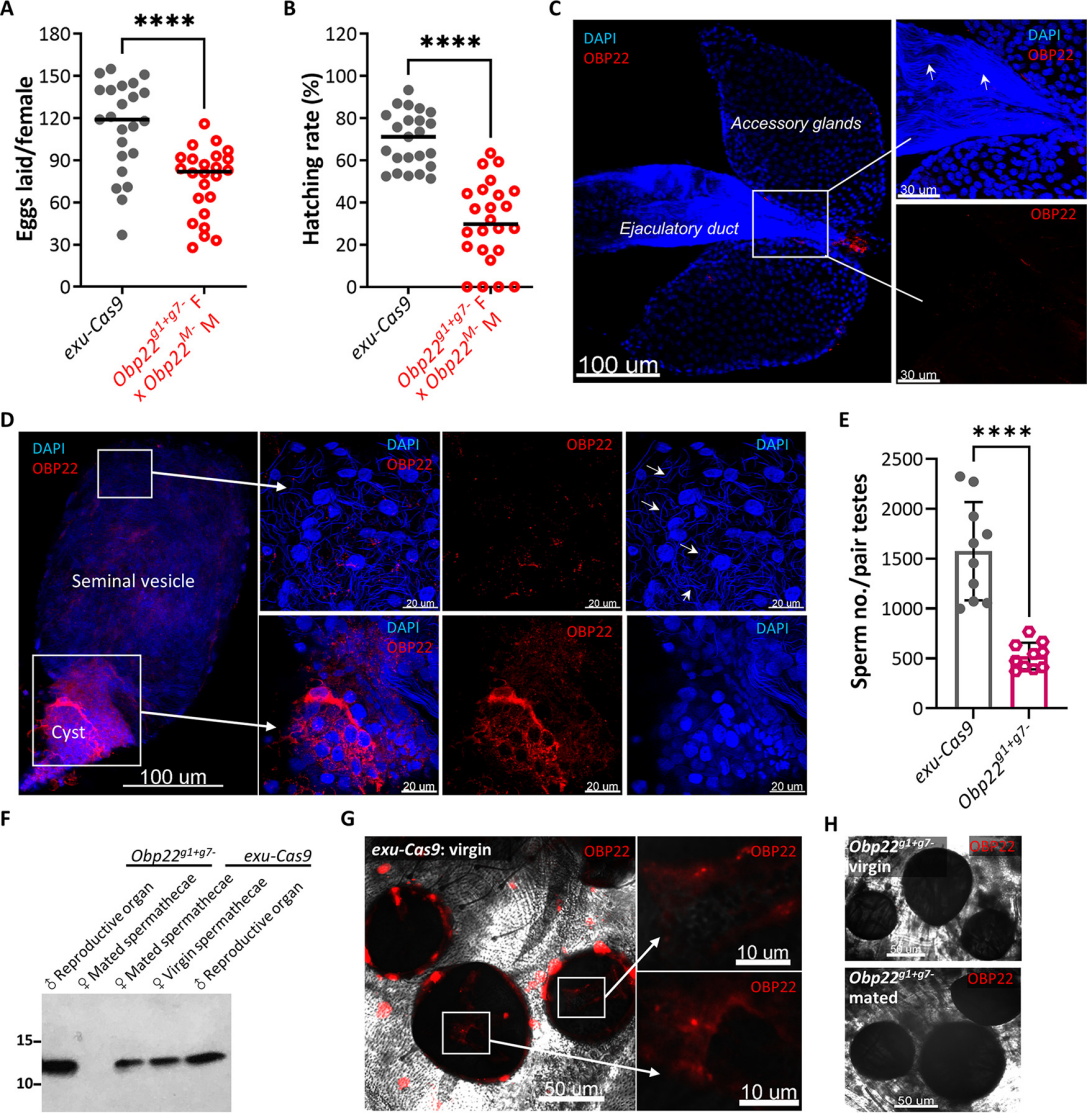
OBP22 affects male fertility by regulating the development of spermatozoa. (A and B) The number of eggs laid by *Obp22*-null mutants (*Obp22^M−^* × *Obp22^g1+g7−^*) and their hatching rate. The *exu-Cas9* line was used as a control. (C and D) IFA detection of OBP22 in male accessory glands and testes of *exu-Cas9* males using OBP22 polyclonal antibody (red). The nuclei were stained with 4′,6-diamidino-2-phenylindole (DAPI; blue). The white arrow indicates spermatozoa in the testes. (E) The number of sperm in the testes of *Obp22^g1+g7−^* and *exu-Cas9* males. (F) Western blot detection of OBP22 in the male reproductive apparatus and female spermathecae collected from *exu-Cas9* and *Obp22^g1+g7−^* mosquitoes. The numbers on the left are the sizes of the molecular standards. (G and H) IFA detection of OBP22 in spermathecae of *exu-Cas9* and *Obp22^g1+g7−^* females using OBP22 polyclonal antibody (red). The zoomed areas are indicated as white boxes and arrows. *P* values were determined by using a Mann-Whitney test (A and B) or an unpaired *t* test (E) with GraphPad Prism. *, *P < *0.05; **, *P < *0.01; ***, *P < *0.001; ****, *P < *0.0001.

**(i) Disruption of *Obp22* impairs male fertility.** Since *Obp22* is linked to the M locus, we looked for potential male-specific function(s) of this gene. Also, because the fertility of heterozygous *Obp22* KO male mosquitoes is compromised (Fig. 3F), it appears that OBP22 indeed plays a role in male reproduction. To confirm a role for OBP22 in male reproduction, we crossed *Obp22* KO male null mutant (*Obp22^M−^*) mosquitoes with homozygous *Obp22^g1+g7−^* females and compared their fecundity and fertility to those of *exu-Cas9* controls. We found that both the numbers of eggs laid by the *Obp22^M−^* × *Obp22^g1+g7−^* mosquitoes and their egg-hatching rates were significantly lower than those of the *exu-Cas9* controls (Fig. 4A and B). These results collectively demonstrate that *Obp22* plays a significant, but not essential, role in male fertility.

**(ii) OBP22 is produced in testes and regulates the development of spermatozoa.** To determine whether OBP22 is produced in the male reproductive apparatus, we performed IFA and Western blotting with OBP22 polyclonal antibodies. IFA showed that OBP22 was highly expressed in the testes but not in the accessory glands or ejaculatory duct of *exu-Cas9* mosquitoes (Fig. 4C and D). In the testes, OBP22 was highly expressed in the apical stem cells within the spermatogonium cyst (Fig. 4D, lower panels) and weakly expressed in testicular follicle cells in the seminal vesicles (Fig. 4D, upper panels), suggesting that OBP22 may mediate the development and maturation of the spermatozoa. This hypothesis was verified by showing that the quantity of sperm was significantly lower in the testes of heterozygous *Obp22^g1+g7−^* males than that in *exu-Cas9* males (Fig. 4E). In addition, OBP22 was not detected on the surface of the spermatozoa in the seminal vesicles (Fig. S3), suggesting that it does not bind to spermatozoa to regulate their development or movement in the testes. The expression of OBP22 in the testes was further validated by Western blotting, and one specific band for OBP22 was detected in the male reproductive tissues of both the *exu-Cas9* and heterozygous *Obp22^g1+g7−^* mosquitoes (Fig. 4F).

10.1128/mBio.02531-21.3 FIG S3 OBP22 is not expressed on the surface of spermatozoa. IFA detection of OBP22 in testes of *exu-Cas9* males. OBP22 was stained with its polyclonal antibody (red), and nuclei were stained with 4′,6-diamidino-2-phenylindole (DAPI; blue). Download FIG S3, PDF file, 0.7 MB. Copyright © 2021 Dong et al. 2021 Dong et al. https://creativecommons.org/licenses/by/4.0/ This content is distributed under the terms of the Creative Commons Attribution 4.0 International license.

**(iii) OBP22 is not required for the fertilization of eggs.** Since OBP22 is apparently produced in the male reproductive system, we next asked whether OBP22 was transferred from males to females during mating. Spermathecae from the virgin females and those mated within 1 day were dissected and used for the detection of OBP22 by IFA and Western blotting. IFA results showed that OBP22 was expressed in the spermathecae of both virgin and mated *exu-Cas9* females (Fig. 4G and Fig. S4), but not in those of virgin and mated *Obp22^g1+g7−^* females (Fig. 4H and Fig. S4), suggesting that OBP22 was not transferred from males to females during mating. This conclusion was also corroborated by the observation that OBP22 was not detected in the accessory glands or ejaculatory duct of *exu-Cas9* (Fig. 4C) males. In addition, a specific OBP22 band was detected in spermathecae of the virgin and mated *exu-Cas9* females, whereas there was no OBP22 band detected in the spermathecae of the virgin and mated *Obp22^g1+g7−^* females by Western blotting (Fig. 4F). These data collectively demonstrate that OBP22 is not injected into females during mating and therefore is not required for female fertility.

10.1128/mBio.02531-21.4 FIG S4 IFA detection of OBP22 in spermathecae of *exu-Cas9* (A) and *Obp22^g1+g7−^* females (B) that mated with *exu-Cas9* males using OBP22 polyclonal antibody (red). Zoomed areas are indicated by white boxes and arrows. Download FIG S4, PDF file, 0.4 MB. Copyright © 2021 Dong et al. 2021 Dong et al. https://creativecommons.org/licenses/by/4.0/ This content is distributed under the terms of the Creative Commons Attribution 4.0 International license.

Taken together, our data show that OBP22 contributes to male fertility by affecting the development of spermatozoa in testes and that this function impacts but is not required for the fertilization of eggs.

### Disruption of *Obp10* or *Obp22* reduces flavivirus transmission by affecting feeding propensity and viral shedding into the saliva.

**FIG 5:**
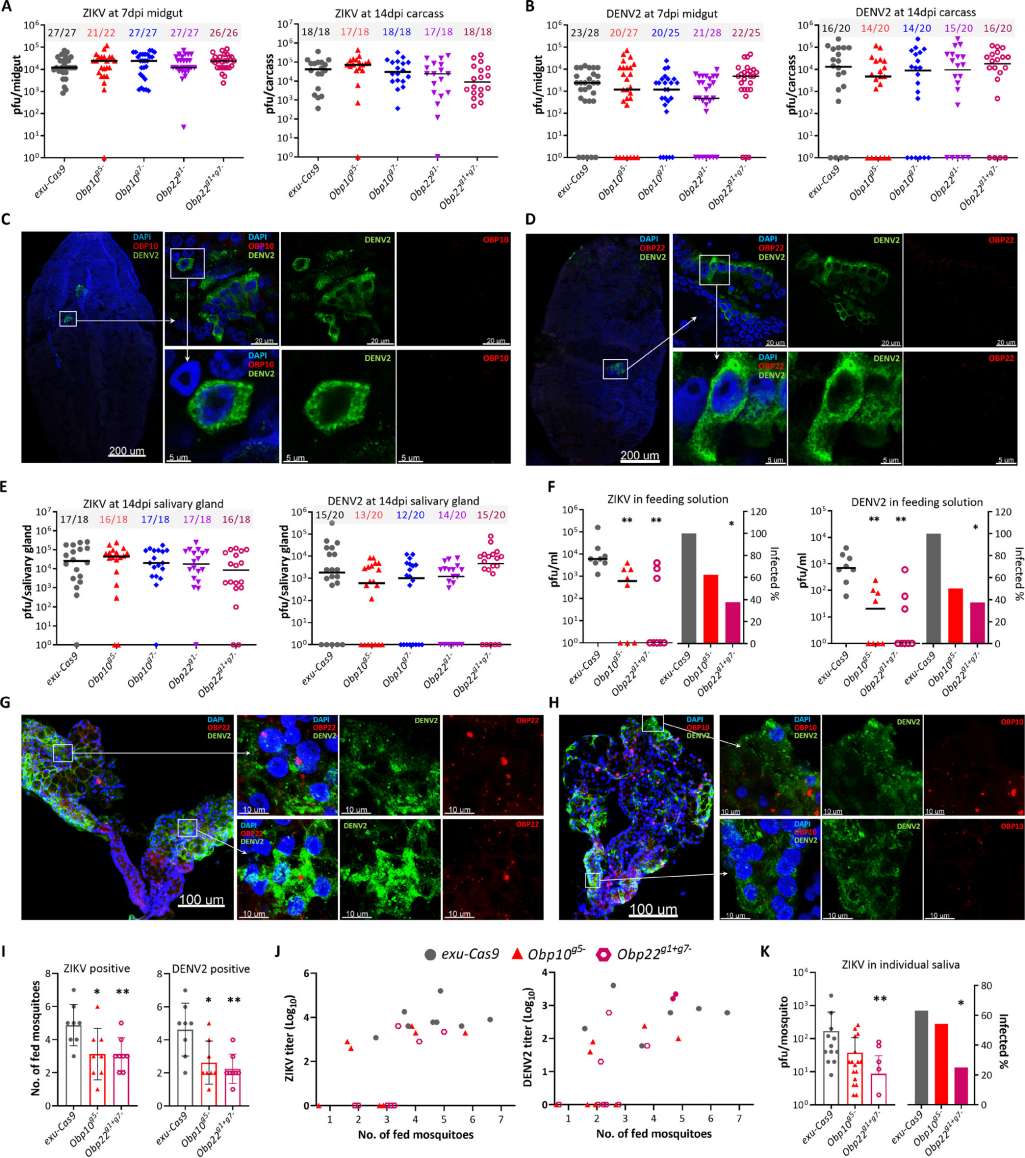
Disruption of OBP10 or OBP22 reduces DENV2 and ZIKV transmission, but does not affect viral infection in A. aegypti. (A and B) The viral infection intensity (virus titer) and infection prevalence (numbers on top of the graphs indicate infected/all tested mosquitoes) in the midguts of females at 7 days postinfection (dpi) and carcasses at 14 dpi. Horizontal lines indicate medians. (C and D) IFA detection of DENV2 and OBP10 or OBP22 in the midguts of *exu-Cas9* females at 7 dpi with the corresponding OBP polyclonal antibody (red) and DENV2 monoclonal antibody (green). Nuclei were stained with DAPI (blue). (E) Viral infection intensity and infection prevalence in salivary glands at 14 dpi. (F) *In vitro* assay of viral transmission by females at 14 dpi. (G and H) IFA detection of DENV2 and OBP10 or OBP22 in salivary glands of *exu-Cas9* females at 14 dpi. (I) Number of fed mosquitoes in each cup. (J) Virus titer plots against the number of fed mosquitoes in each cup. (K) Virus titer of individual saliva samples. Statistical significance was determined by a Mann-Whitney test for infection intensity or a Fisher’s exact test for infection prevalence. *, *P < *0.05; **, *P < *0.01.

**(i) OBP10 and OBP22 are not required for flavivirus infection and replication but affect virus transmission by mosquitoes.** To assess the effect of *Obp10* or *Obp22* disruption on flaviviral infection and dissemination in mosquitoes, we fed a pool of homozygous *Obp* mutants, along with WT control (*exu-Cas9*) mosquitoes, on DENV2- or ZIKV-containing blood, and then used plaque assays to measure virus titers in the midguts at 7 days postinfection (dpi) and in carcasses at 14 dpi. Only fully engorged mosquitoes were selected to minimize the effect of feeding impairment by *Obp* KO. Compared to control mosquitoes, *Obp10* or *Obp22* disruption did not exert a significant impact on either the infection intensity or prevalence of both DENV2 and ZIKV in 7-dpi midguts and 14- dpi carcasses (Fig. 5A and B). Thus, neither *Obp10* nor *Obp22* appeared to influence flavivirus replication or infection in A. aegypti. We then used IFA to examine OBP10 and OBP22 expression in midguts with DENV2 at 7 dpi, and the results showed that neither OBP10 nor OBP22 was expressed in the midguts of the *exu-Cas9* females, whereas their midgut cells were consistently infected with DENV2 (Fig. 5C and D). These data reveal that OBP10 and OBP22 are neither involved nor required for flaviviral infection and dissemination in the mosquito midgut.

To determine whether the ability of the mosquitoes to transmit flavivirus was in some way influenced by either of the two *Obp* genes, we first examined the effect of *Obp* KO on virus production in the salivary glands. The results showed that there were no significant differences between control and *Obp* KO females in the infection intensity and prevalence of either DENV2 or ZIKV in the salivary glands at 14 dpi (Fig. 5E). We then examined the levels of virus particles released from the proboscis during feeding by *Obp10^g5−^* or *Obp22^g1+g7−^* females and *exu-Cas9* females. At 14 dpi, a group of mosquitoes (8 females/cup) were fed on a feeding solution (50% human serum in Dulbecco’s modified Eagle’s medium [DMEM]) though a sterile glass membrane feeder for 60 min, and titers of the viruses in the solution were then determined by plaque assay. Compared to the control females, *Obp10* and *Obp22* KO mosquitoes both released a significantly lower number of ZIKV and DENV2 particles from their saliva into the feeding solution (Fig. 5F). Infectious virus particles were detected in the feeding solution from all eight groups of control females, whereas the feeding solutions of only 5 and 4 groups of *Obp10* KO females contained ZIKV or DENV2 particles, respectively. In *Obp22* KO females, only 3 groups had ZIKV- or DENV2-positive particles, and the numbers were significantly lower than those of the *exu-Cas9* females (Fig. 5F). Thus, our data demonstrate that *Obp10* and *Obp22* do not influence flavivirus production in salivary glands, but they do impact the transmission potential of the flaviviruses.

**(ii) OBP22 facilitates viral shedding into the saliva.** To gain insight as to why *Obp10* or *Obp22* disruption affects flaviviral transmission, we first conducted IFA to look for colocalization of OBP10 or OBP22 with DENV2 in salivary glands at 14 dpi. IFA showed that OBP10 and OBP22 were expressed in DENV2-infected salivary glands of *exu-Cas9* females (Fig. 5G and H). DENV2 particles were also detected in the salivary glands of *Obp10^g5−^* or *Obp22^g1+g7−^* females, but there was no signal for either OBP10 or OBP22 (Fig. S5). A strong DENV2 signal was detected in the restricted area/compartment of the salivary glands of *Obp22^g1+g7−^* females (Fig. S5), suggesting that these virus particles may be prevented from being released from the salivary gland cells in the absence of *Obp22*.

10.1128/mBio.02531-21.5 FIG S5 Salivary glands of *Obp* knockout (KO) mosquitoes are infected by dengue virus serotype 2 (DENV2). Salivary glands of *Obp22^g1+g7−^* (A) and *Obp10^g5−^* (B) females at 14 days postinfection (dpi) were stained with their corresponding polyclonal antibody (red) and DENV2 monoclonal antibody (green). The nucleus was stained with DAPI (blue). Zoomed areas are indicated by white boxes and arrows. Download FIG S5, PDF file, 0.4 MB. Copyright © 2021 Dong et al. 2021 Dong et al. https://creativecommons.org/licenses/by/4.0/ This content is distributed under the terms of the Creative Commons Attribution 4.0 International license.

Next, we counted the number of blood-fed mosquitoes in each group in order to determine whether virus-infected KO mutants display any altered feeding propensity. We found that the number of fed ZIKV- and DENV2-infected *Obp10* or *Obp22* KO mosquitoes was significantly lower than that for the *exu-Cas9* females (Fig. 5I). Interestingly, we were unable to detect any positive DENV2 or ZIKV particles in the feeding solution, although a few *Obp* KO mosquitoes fed on these solutions (Fig. 5J), suggesting that the virus particles were not released into the feeding solution through the saliva, or that the salivary glands were not infected. We then compared infectious viral particles in the saliva of individual *Obp* KO and control female mosquitoes using a forced-salivation method. Our results showed no significant differences in ZIKV titer or infection prevalence in the saliva of the *Obp10* KO and control mosquitoes; however, the *Obp22* disruption significantly reduced the ZIKV titer and infection prevalence in the saliva (Fig. 5K). These data suggest that the reduced transmission of viruses results from an impaired feeding capacity in *Obp10* KO mosquitoes, whereas both an impaired feeding capacity and a failure to release viral particles into the saliva account for the significantly low transmission of flaviviruses by the *Obp22* KO mosquitoes.

In summary, our data show that OBP10 and OBP22 are not required for virus infection or replication in mosquito midguts and salivary glands, but they do influence flaviviral transmission by affecting feeding propensity and the shedding of flaviviral particles into the saliva.

***Obp10* or *Obp22* disruption reduces female longevity but does not affect larval development or the sex ratio.** Other fitness parameters, including larval development, sex ratio, and longevity, were compared between the *Obp* KO and the control (*exu-Cas9*) mosquitoes. The larval development and sex ratios were not affected by KO of *Obp10* or *Obp22* (Fig. S6A and B); however, the longevity of the females (Fig. S6C), but not that of the males (Fig. S6D), was significantly reduced when the mosquitoes were fed on a regular sugar meal. The longevity of the *Obp22* KO females was also significantly reduced when they were fed on a blood meal (Fig. S6E).

10.1128/mBio.02531-21.6 FIG S6 Effects of *Obp10* or *Obp22* mutation on larval development, sex ratio, and longevity. Pupation time (A) and sex ratio (B) did not differ between the *Obp* knockout (KO) and the control (*exu-Cas9*) mosquitoes. Statistical significance was determined by the Gehan-Breslow-Wilcoxon test (A) or unpaired *t* test (B). Longevity was significantly decreased in *Obp* KO females (C), but not in *Obp* KO males (D), when mosquitoes were fed on 10% sugar. (E) The longevity of *Obp22* KO females also significantly decreased after they received a naive blood meal. The *P* value was determined by the log rank (Mantel-Cox) test. Download FIG S6, PDF file, 0.3 MB. Copyright © 2021 Dong et al. 2021 Dong et al. https://creativecommons.org/licenses/by/4.0/ This content is distributed under the terms of the Creative Commons Attribution 4.0 International license.

## DISCUSSION

Beyond their canonical roles in olfaction, accumulating evidence points to a wide range of pleiotropic functions for insect OBPs and, with the exception of those of a few Drosophila OBPs (28, 29), those cryptic functions have not been well defined, primarily due to a lack of functional genetics approaches for eliminating these proteins *in vivo*. Here, we have knocked out the function of two OBPs in *A. aegypti* and uncovered their roles in female and male reproduction and arbovirus transmission, thus extending their utility beyond chemosensation.

Together with our previous study (25), our data demonstrate that OBP10 and OBP22 play notable, but nonessential, roles in host- seeking by affecting blood-feeding behavior and intake in A. aegypti, since the impact of these mutations on blood feeding is only moderate. This assertion is further strengthened by our studies of EAG responses to key odorants, which failed to indicate any significant differences in antennal sensitivity between *Obp10* or *Obp22* KO and *exu-Cas9* proxy-wild-type mosquitoes. Consistent with these data, KO of a single highly expressed *Obp* (*Obp28a*) in *Drosophila* does not reduce the magnitude of its responses to a diverse panel of odorants (29). These data support a model in which host seeking, oviposition, and other mosquito behaviors are regulated by multiple OBPs and depletion of the function of one OBP function can likely be complemented by those of other OBPs. This potential complementation may also explain the large number of 111 *Obp* genes in the A. aegypti genome (17, 19).

Depletion of OBP10 or OBP22 significantly reduces female fecundity and fertility. Our data support that mutation of *Obp10* or *Obp22* reduces the size of the blood meal taken by females, leading to the decreased expression of trypsin genes and subsequent trypsin activity, thereby affecting protein digestion, which results in the reduced expression of reproductive genes. As a result, the number of eggs and their hatching rate are decreased. Thus, depletion of OBP10 or OBP22 significantly impacts female reproduction, and the impact occurs through affecting blood-feeding capability.

A few OBPs have been localized in the male reproductive organs (30–32), but their molecular and physiological functions remain speculative. Here, we present a number of lines of evidence to indicate that OBP22 plays an important role in male reproduction in A. aegypti, likely by influencing the development of spermatozoa in the testes. We found that (i) *Obp22* is linked to the male-determining sex locus (M) on chromosome 1, (ii) the fertility of *Obp22* male null mutants was significantly lower than that of the controls, (iii) OBP22 was highly expressed in the spermatogonial cyst of the testes, and (iv) the quantity of sperm was significantly decreased after disruption of OBP22.

A model in which OBP22 is involved in the development of spermatozoa in male testes is congruent with a recent study in which the odorant receptor coreceptor, *Orco*, was demonstrated to be expressed in the spermatozoa of several insect species, including A. aegypti and the malaria mosquito Anopheles gambiae, and to be involved in the activation of the spermatozoa in the male testes and female spermatheca (32). However, unlike *Orco* in mosquitoes (32), OBP22 is not directly linked to spermatozoa to directly regulate spermatogenesis, suggesting that it may instead serve as a small-molecule carrier of endogenous pheromones that mediate the development of spermatozoa. This hypothesis is supported by the findings from a recent study in which one member of the OBP family (*mJHBP*) was found to specifically bind to juvenile hormone as a potential means of regulating endocrine signaling in A. aegypti (33). This scenario is common in Lepidoptera, in which pheromone-binding proteins (PBPs), a subfamily of OBPs, play an important role in recognizing and transporting pheromones and host odorants (34). Chemosensory proteins (CSPs), which are functionally related to OBPs, have also been shown to be expressed in pheromone glands and may bind and transport pheromone molecules in the cabbage moth Mamestra brassicae (35). Thus, we speculate that OBP22 may serve as an endogenous pheromone or endocrine signal chaperone or carrier.

It has been shown that the seminal fluid in the male accessory glands can serve as a transport, activation, and nourishing medium for sperm (36). However, we did not detect OBP22 in the male accessary glands or ejaculatory ducts, suggesting that OBP22 is not transferred to the female during copulation. This hypothesis is further supported by the finding that OBP22 is not detected in spermathecae from either virgin or mated *Obp22* KO females by Western blotting or IFA, indicating that OBP22 is not required for maintaining the sperm mobility that is necessary for sperm transfer to females during copulation. Our finding contradicts a previous study in which the authors suggested that OBP22 could be transferred to females (31). This discrepancy may have occurred because OBP22 is expressed in the spermathecae of both virgin and mated WT females, which could have caused the strong OBP22 signal observed in their study (31), rather than being a result of mating.

To complete the infection cycle between the mosquito vector and its vertebrate host, arboviruses must be shed into the saliva after replication in the mosquito’s salivary gland cells. Thus far, the mechanisms involved in viral shedding into the saliva have yet been determined, but in mammalian cells, mature virions are released by exocytosis after transport to the plasma membrane in vesicles (37). *Obp22* has been found to be upregulated in the salivary gland of mosquitoes infected with DENV2, suggesting the importance of OBP22 in mosquito-arbovirus interaction (25). In our study, disruption of OBP22 resulted in reduced viral titer and prevalence in the saliva, but not in the salivary glands, and viral particles were restricted to the area/compartment of the salivary glands in the *Obp22* KO mosquitoes, indicating that OBP22 plays a role in facilitating efficient flavivirus secretion into the saliva. It remains unclear how OBP22 facilitates viral shedding from the host cells. It is possible that OBP22 binds to specific ligands that mediate cellular signaling to facilitate virus egression/secretion; this hypothesis is supported by the fact that OBP22 is able to bind to multiple ligands, including fatty acids (26, 27, 31), but further studies are obviously required to test this hypothesis.

Reduction of the virus transmission potential of mosquitoes is critical for controlling mosquito-borne arboviral diseases. Our data show that OBP10 and OBP22 do not play a role in flavivirus infection or replication in mosquito midgut and salivary gland cells. However, given the impairment of blood feeding that occurs in response to *Obp10* or *Obp22* disruption, the overall infection prevalence and intensity are affected by the KO. Moreover, the potential for virus transmission is significantly reduced in *Obp* KO mosquitoes. Coupled with the impairment of host-seeking behavior and the shortened life span of *Obp* KO females, our data suggest that the vectorial capacity should be remarkably reduced in *Obp*-impaired mosquitoes. Thus, OBPs appear to represent potent flavivirus transmission-blocking targets.

## MATERIALS AND METHODS

### Ethics statement.

This study was carried out in accordance with the recommendations in the Guide for the Care and Use of Laboratory Animals of the National Institutes of Health, the Animal Care and Use Committee (ACUC) of the Johns Hopkins University, and the institutional Ethics Committee (permit number M006H300). The Institutional Animal Care and Use Committee (IACUC) approved the protocol. Mice were only used for mosquito rearing. Commercial, anonymous human blood was used for virus infection assays in mosquitoes, and informed consent was therefore not applicable.

### Mosquitoes.

The germ line Cas9-expressed A. aegypti line (*exu-Cas9*) gifted to us by Omar Akbari was reared and maintained at 27°C under 85% relative humidity and a 12-h light/12-h dark cycle. All of the *Obp* knockout mosquitoes were reared under the same conditions in a walk-in chamber in the insectary of Johns Hopkins Malarial Research Institute. Mice were used for blood feeding and colony maintenance.

### gRNA design and synthesis.

The guide RNA (gRNA) targets of *Obp* were designed using the chopchop CRISPR/Cas9 tool (https://chopchop.cbu.uib.no/). To efficiently mutate the *Obp* genes, multiple gRNAs targeting *Obp10* or *Obp22* exons were designed (see Table S2 in the supplemental material). To increase the possibility of two gRNAs working concurrently and inducing a large deletion, we selected multiple gRNAs targeting the same 300-bp DNA sequence for each *Obp*.

10.1128/mBio.02531-21.9 TABLE S2 List of primers. Download Table S2, PDF file, 0.5 MB. Copyright © 2021 Dong et al. 2021 Dong et al. https://creativecommons.org/licenses/by/4.0/ This content is distributed under the terms of the Creative Commons Attribution 4.0 International license.

The double-stranded DNA templates for each gRNA were generated by template-free PCR annealing of the two overlapping primers using high-fidelity *Taq* DNA polymerase (Thermo Fisher Scientific). The reverse primer CRISPR_R is universal for all of the single guide RNA (sgRNA templates), and the forward primer CRISPR_F included a T7 promoter, the specific gRNA target sequence, and the overlapping sequence (38). The PCR conditions were as follows: heating to 95°C for 30 s; followed by 35 cycles at 95°C for 10 s, 58°C for 10 s, and 68°C for 10 s; then 68°C for 5 min. The PCR products were purified using a DNA Clean & Concentrator kit (Zymo Research) and then *in vitro* transcribed using a HiScrib T7 Quick high-yield RNA synthesis kit (NEB). Following transcription, the gRNAs were purified using an RNeasy kit (Qiagen), eluted with nuclease-free water, and diluted to 1 μg/μl for long-term storage at −80°C. The quality of the purified gRNAs was evaluated on agarose gels.

### Embryo microinjection and screening for *Obp* KO lines.

For injection of mosquito embryos, each gRNA was diluted to 100 ng/μl with E-Toxate water (Sigma) and filtered through a 0.2-μm, 1.5-ml filter (Millipore). The mosquito embryo microinjection was done according to our previously established protocol (39). For each gRNA or gRNA mix, 200 to 400 *exu-Cas9* eggs were injected.

A leg PCR was used to screen the surviving G0 adults for mutations using a pair of primers outside ∼100 bp of each gRNA (Table S2). In brief, one hind leg of the adult mosquito was pulled and transferred to a PCR tube with 20 μl Phire reaction buffer (Thermo Fisher Scientific), then heated at 95°C for 5 min, and 1 μl was used as a template for PCR amplification. PCR mixtures were heated to 95°C for 2 min; followed by 40 cycles at 95°C for 10 s, 58°C for 20 s, and 68°C for 30 s. PCR products were checked on a 2% agarose gel, and an extra band was considered to indicate a mutation induced by gRNA. The mutated PCR products were purified using a DNA Clean & Concentrator kit (Zymo Research), sequenced, and compared to the WT gene to confirm the mutation. The mutated G0 mosquito was then outcrossed with *exu-Cas9* mosquitoes. After outcrossing of the *Obp* mutants with *exu-Cas9* mosquitoes for several generations, heterozygous mutants were incrossed, and the mutated homozygotes were identified by leg PCR and confirmed by DNA sequencing.

### Generating homozygous *Obp22* mutant males.

*Obp22* is tightly linked to the dominant male-determining sex locus (M) on chromosome 1 (Fig. S7A) (40). Both *Obp22^g1−^* and *Obp22^g1+g7−^* were first generated from G0 females. Thus, only the m-linked *Obp22* can be mutated. As a result, all of the *Obp22* KO (both *Obp22^g1−^* and *Obp22^g1+g7−^*) males were heterozygous, with both the M-linked WT inherited from the father and the m-linked truncated *Obp22* alleles inherited from the mother. M-linked *Obp22* mutation was generated from a cross between G0 KO males and *exu-Cas9* females. G1 males were PCR screened and sequenced, and those males with the same mutation as *Obp22^g1+g7−^* were crossed with *Obp22^g1+g7−^* females. From this cross, *Obp22* KO homozygous males (*Obp22^M−^*) were obtained and confirmed by leg PCR and Sanger sequencing (Fig. S7B).

10.1128/mBio.02531-21.7 FIG S7 (A) *Obp22* is predicated to be linked to a sex-differentiated region that includes a dominant male-determining sex locus (M) on chromosome 1. (B) PCR amplification of *Obp22* in male homozygous mutants (*Obp22^M−^*) and sequencing trace data. Download FIG S7, PDF file, 0.6 MB. Copyright © 2021 Dong et al. 2021 Dong et al. https://creativecommons.org/licenses/by/4.0/ This content is distributed under the terms of the Creative Commons Attribution 4.0 International license.

### Blood feeding and endogenous trypsin activity assay.

Five 1-week-old females were transferred to a small cup (16-oz Solo) or a cage (length = 8 in., width = 8 in., height = 8 in.) and starved for 4 to 6 h, then fed at around 2 p.m. on an anesthetized mouse for 5 min or 20 min. The blood-fed mosquitoes in each cup or cage were determined as partly and fully engorged and counted. Mosquitoes were anesthetized with carbon dioxide, and the weight of every five mosquitoes was measured on a fine balance (Ohaus). These five mosquitoes were placed in a small cup for blood feeding on a mouse. After blood feeding, mosquitoes were anesthetized and weighed as described above. The blood meal size for five mosquitoes was calculated by subtracting the weight of the mosquitoes before blood feeding from the weight afterwards. All of the assays were performed with five technical repeats and at least two biological replicates. Data were pooled from different replicates and used for generating graphs.

Six midguts per group from mosquitoes at 1 or 2 days post blood meal (PBM) were dissected on ice and then homogenized in 120 μl of cold reaction buffer (50 mM Tris-HCl [pH 8.0] with 10 mM CaCl_2_) with a pestle. Supernatants were collected after centrifugation at 20,000 × *g* at 4°C for 10 min. Trypsin activity assays were performed using the synthetic colorimetric substrate *N*α-benzoyl-d,l-arginine-*p*-nitroanilide hydrochloride (BApNA; Sigma) as described previously (41). The reaction mixtures contained 2 μl (or 10 μl) of midgut extracts of mosquitoes at 1 day (or 2 days) PBM and 1 mM BApNA in a final 100 μl of reaction mixture. Absorbance values were measured as optical density at 405 nm (OD_405_) every 5 min for 30 min using a plate reader. At least five technical repeats were performed, with three biological replicates. Data from one biological replicate were used for generating the graph.

The statistical significance (expressed as *P* value) of the differences between the *Obp* KO lines and the control *exu-Cas9* line was determined by using an unpaired *t* test with GraphPad Prism.

### Fecundity, hatching rate, and mating.

One-week-old *Obp* KO and *exu-Cas9* females were fed on mice, and fully engorged mosquitoes were sorted. At 3 days PBM, individual females were each placed in a 50-ml conical tube with moist filter paper at the bottom of the tube; at least 25 females were included for each line, and at least two biological replicates were performed. After 24 h, the egg papers were removed from the tube and photographed. The number of eggs on the filter paper was counted using ImageJ software. The eggs laid by each individual female were hatched in DI water at 4 days post egg laying, and larvae were counted 2 days after hatching. The hatching rate was calculated by dividing the number of larvae by the number of eggs laid per female. Dot-plotted graphs were generated using GraphPad Prism, and the *P* values between *Obp* KO females and *exu-Cas9* females were determined by using a nonparametric Mann-Whitney test.

### Oviposition.

At 3 days PBM, five females were placed in a cage (length = 8 in., width = 8 in., height = 8 in.) containing an oviposition cup with moist filter paper. After 16 h, the egg papers were removed from the oviposition cup and photographed. The number of eggs on the filter was counted using ImageJ software. The ovaries were dissected from females, and mature eggs remaining in the ovaries were recorded; any case in which the female had fewer than 10 eggs remaining in the ovary was considered a successful oviposition. The oviposition percentage was calculated by dividing the number of successfully ovipositing females by five. Three technical repeats were performed, with three biological replicates. Graphs showing individual dots for each female were generated using GraphPad Prism, and the *P* values between *Obp* KO females and *exu-Cas9* females were determined by using a nonparametric Mann-Whitney test.

### Larval development, pupation time, and adult survival rate.

Fifty *Obp* KO and *exu-Cas9* first-instar larvae were reared in a container with fish food according to a standard procedure, and the number of pupae was recorded each day to determine the development time. Each line had at least three biological replicates, with three technical repeats.

Adults were placed into cups after emergence, and 10% sterile sucrose solution was provided with a cotton pad that was changed every 2 days. To investigate the survival percentage of blood-fed mosquitoes, 5-day-old females were fed on mice, and fully engorged females were transferred to a new cup. Each cup had 20 females, and there were at least three replicates for each line. The number of dead mosquitoes in each cup was recorded, and dead mosquitoes were removed daily. Statistical significance was determined by Kaplan-Meier survival analysis with pooled data from three replicates by using GraphPad Prism software, and the *P* values were determined by the Gehan-Breslow-Wilcoxon test.

### Electrophysiological recording.

The electroantennogram (EAG) procedure was modified on the basis of a previous study (42). In brief, a 5- to 10-day-old female mosquito was decapitated with forceps. Two sharp borosilicate glass (1B100F-3; World Precision Instruments, Sarasota, FL) electrodes were prepared using an electrode puller (P-97; Sutter Instruments, Novato, CA) and filled with 0.1 M KCl. After the tip of the reference electrode was broken to make a suitable opening, the electrode was inserted into the back of the mosquito’s head, and the recording electrode was placed in contact with the cuticle at the tip of the antenna. Antennal preparations were continuously exposed to a humidified airflow (1.84 liters/min) transferred through a borosilicate glass tube (inner diameter = 0.8 cm) that was exposed to the preparation at a distance of 10 mm. A 10-μl aliquot of odorant solution was placed on a piece of filter paper (3 mm × 50 mm), which was then inserted into a 6-in. Pasteur pipette to create the stimulus cartridge. The stimulus was delivered to antennal preparations with a 0.5-s air pulse through a hole placed on the side of the glass tube located 10 cm from the open end of the delivery tube (1.08 liters/min), where it was mixed with the continuous airflow using a dedicated stimulus controller (Syntech, Hilversum, The Netherlands). Airflow (0.76 liters/min) was simultaneously delivered from another valve through a blank pipette into the glass tube at the same distance from the preparation in order to minimize changes in flow rate during odor stimulation. A sample containing the solvent alone served as the negative control. The response data were collected with a 4-channel Intelligent Data Acquisition Controller (IDAC)-USB signal acquisition instrument (Syntech GmbH, Buchenbach, Germany), and analyzed with EAG2000 software (Syntech) on a personal computer. The raw odor responses were divided by the solvent responses to generate normalized EAG responses. Odorants were diluted with paraffin oil (Sigma-Aldrich, St. Louis, MO) to make 10^−1^ (vol/vol) working solutions, except for l-(+)-lactic acid and ammonia, which were diluted with deionized water; phenol, indole, and skatole were diluted with dimethyl sulfoxide (DMSO) (Sigma-Aldrich). *P* values between the *Obp* KO and the control *exu-Cas9* line were determined by using an unpaired *t* test with GraphPad Prism.

### Virus infection and transmission in mosquitoes and plaque assay.

ZIKV or DENV2 infection of mosquitoes and plaque assays was performed as described previously (43). ZIKV (Cambodia, FSS13025) or DENV serotype 2 (DENV2; New Guinea C strain) was propagated in C6/36 cells. The virus-infected cell culture medium was harvested and mixed with an equal volume of commercial human blood supplemented with 10% human serum containing 10 mM ATP. One-week-old *Obp* KO and *exu-Cas9* females were starved overnight and transferred into a small cup, then fed for 30 min with the blood-virus mixture at 37°C on a single glass artificial feeder. Fully engorged females were selected and maintained on 10% sterile sucrose solution. Midguts from 7-dpi individual females and carcasses and salivary glands from 14-dpi individual females were dissected on ice and kept it at −80°C. Each experiment had two biological replicates, and each replicate included at least 18 individual females.

Mosquito midguts, carcasses, or salivary glands were homogenized in 300 μl DMEM with a Bullet Blender (Next Advance, Inc., Averill Park, NY) with glass beads. They were then centrifuged at 10,000 × *g* for 3 min, and 50 μl of the filtered supernatants was used for plaque assays with BHK 21 cells in 24-well plates. The plates were incubated for 4 days for ZIKV and 5 days for DENV2, and then the plaques were visualized by staining with 1% crystal violet and counted under a microscope.

Viral transmission in mosquitoes was assayed using an artificial glass feeder (44). At 13 dpi, eight mosquitoes were transferred to a small cup (4 cups/line). On the second day, mosquitoes were fed on a sterilized glass feeder with 200 μl of feeding solution (equal volumes of human serum and DMEM with a final 10 mM concentration of ATP) for 1 h. The feeding solution from each feeder was collected in a PCR tube and titer was determined using a plaque assay. The fed mosquitoes were counted on ice under the microscope. Two biological replicates were performed, and the data were pooled from different replicates and used for generating graphs.

The saliva from individual females was collected using forced salivation. On ice, the legs and wings were carefully removed from the mosquitoes. The fine ends of 10-μl pipette tips were cut, and the tips filled with 10 μl of feeding solution. Each mosquito proboscis was placed into the end of a pipette tip and left for 1 h at room temperature (RT). The mosquito was then removed, and the tips were placed in individual PCR tubes with 50 μl of sample processing buffer (10% fetal bovine serum [FBS] and 1% penicillin-streptomycin in minimal essential medium [MEM]), and infectious virial particles in the saliva samples were determined by plaque assay. Each experiment had two biological replicates, and each replicate included at least eight individual females. Data were pooled from different replicates and used for generating figures.

Differences in virus intensity and prevalence of infection in samples between *Obp* KO and *exu-Cas9* females were compared by using a nonparametric Mann-Whitney U test and Fisher’s exact test, respectively.

### Reverse transcription-quantitative PCR.

Groups of six *exu-Cas9* or *Obp* KO mosquitoes at 12 days PBM were collected for total RNA extraction with TRIzol reagent (Invitrogen, Carlsbad, CA). First-strand cDNA was synthesized from 0.8 μg total RNA using a QuantiTect reverse transcription kit (Qiagen, Hilden, Germany). Gene-specific primers (Table S2) were used for qPCR amplification of reproductive and trypsin genes. qPCR amplification and analysis were carried out using the StepOnePlus real-time PCR system (Applied Biosystems, Warrington, UK). The final reaction volume was 16 μl when using the ABI SYBR green supermix. The PCR program was as follows: hold at 95°C for 10 min, 95°C for 15 s and 60°C for 1 min, repeated for 40 cycles. The specificity of the SYBR green PCR signal was further confirmed by melting curve analysis. The relative abundance of the gene transcripts was normalized and calculated by comparison to the ribosomal protein *S7* gene (AAEL009496) as an endogenous reference using the comparative threshold cycle (2^−ΔΔ^*^CT^*) method. Each sample had at least three independent biological replicates. The significance (*P* values) of the difference between the results for the *Obp* KO and control *exu-Cas9* line was determined by using an unpaired *t* test with GraphPad Prism.

### Western blot and immunofluorescence assays.

After viral infection, midguts and salivary glands were dissected from females at 7 days postinfection (dpi) or 14 dpi, respectively. Antenna and mouthparts, including the maxillary palps and proboscis, were dissected from 5-day-old uninfected females. Accessory glands and testes were dissected from 5-day-old uninfected males, and spermathecae were dissected from 5-day-old virgin or mated uninfected females. Immunofluorescence assay (IFA) samples were fixed in 4% paraformaldehyde (Sigma) for 1 day to 1 week at 4°C. After permeabilization with phosphate-buffered saline (PBS) containing 1% bovine serum albumin (BSA) and 0.2% Triton X-100 (PBT), the samples were incubated overnight at 4°C with polyclonal antibody against OBP10 or OBP22 that was generated against synthesized OBP10 or OBP22 by Boster Biological Technology Co., Ltd. (Pleasanton, CA). Midgut and salivary gland samples were also incubated with mouse hyperimmune ascitic fluid specific for DENV2 (25). After four washes with PBST (PBS with 0.1% Triton X-100), the samples were incubated with Alexa Fluor 568 goat anti-rabbit IgG (Invitrogen) and Alexa Fluor 488 goat anti-mouse IgG (Invitrogen) in PBT at 37°C in the dark. DAPI (4′,6-diamidino-2-phenylindole) was added to each sample to stain the nuclei. Samples were viewed and photographed under a Zeiss LSM700 confocal microscope at the Johns Hopkins University School of Medicine Microscope Facility.

For Western blotting, samples were homogenized in PBS containing proteinase cocktail (Roche) and centrifuged at 10,000 × *g* for 10 min at 4°C. The supernatants were separated by SDS-PAGE and transferred to a nitrocellulose membrane. After blocking with 5% nonfat milk, the membranes were incubated with OBP10 or OBP22 polyclonal antibody at 4°C. After four washes, the membranes were incubated with anti-rabbit IgG-HRP (Cell Signaling Technology) at room temperature (RT). Chemiluminescent signals were detected by using Amersham ECL Prime Western blotting detection reagents. Anti-β-actin-peroxidase antibody (Sigma) was used to detect the loading control.

### Male sperm quantity and viability assay.

The quantity and quality of male sperm in the testes were checked using a live/dead sperm viability kit (Invitrogen). Testes were dissected from 4-day-old males, and individual pairs were placed into tubes containing 30 μl of PBS. The testes were homogenized gently with a pestle, and another 165 μl of PBS was used to rinse the pestle. A master dye mix was created by combining a 1:1:3 dilution of SYBR 14 (1:10 dilution of stock), propidium iodide, and HEPES buffer (10 mM HEPES, 150 mM NaCl, and 10% BSA [pH 7.4]). To each tube, 5 μl of master dye mix was added, and the mixture was incubated for 5 to 10 min in the dark at RT. A total of 10 5-μl aliquots of sperm dilution were spotted onto a multiwell slide and allowed to dry in the dark at RT (45, 46); 3 μl of PBS was added to each well, which was then coverslipped. The sperm in each sample were counted under a fluorescence microscope. For sperm quality assays, 2-μl aliquots of sperm dilution were spotted onto a multiwell slide and photographed, and the dead/live sperm in each sample were counted under a Zeiss LSM700 confocal microscope at the Johns Hopkins University School of Medicine Microscope Facility. The significance (*P* values) of the difference between the *Obp22* knockout and control *exu-Cas9* lines was determined by using an unpaired *t* test with GraphPad Prism.
