# Supplementary material for: Pleiotropic Odorant-Binding Proteins Promote Aedes aegypti Reproduction and Flavivirus Transmission
Source: mBio. 2021 Oct 12;12(5):e02531-21. doi: 10.1128/mBio.02531-21 (PMC8510553; doi:10.1128/mBio.02531-21)
Supplement: TABLE S1 [file mbio.02531-21-st001.pdf]

Table S1 Data from generating *Obp10* or *Obp22* knockout mosquitoes.

	gRNAs	Eggs injected	Hatched	Hatching rate	Mutated	Mutation rate
Obp10	**gRNA5**	**193**	**103**	**53.4%**	**3 of 6**	**50.0%**
	**gRNA7**	**188**	**99**	**52.7%**	**6 of 6**	**100.0%**
	**gRNA8**	**188**	**128**	**68.1%**	**0 of 6**	**0.0%**
	**gRNA5+gRNA7**	**356**	**170**	**47.8%**	**77 of 94**	**81.9%**
Obp22	**gRNA1+gRNA7**	**308**	**186**	**60.4%**	**39 of 91**	**42.9%**

gRNAs targeting Obp10 or Obp22 were microinjected into eggs of the exu-Cas9 females. The hatching rate was calculated by dividing the number of hatched eggs by the total number of eggs injected for each gRNA or gRNA mix. Mutation of the injected adults (G0) was determined by a leg PCR and/or Sanger sequencing. Mutation rate was calculated by dividing the number of mutated G0 by the total number of G0 surviving for each gRNA or gRNA mix.
