# Supplementary material for: Pleiotropic Odorant-Binding Proteins Promote Aedes aegypti Reproduction and Flavivirus Transmission
Source: mBio. 2021 Oct 12;12(5):e02531-21. doi: 10.1128/mBio.02531-21 (PMC8510553; doi:10.1128/mBio.02531-21)
Supplement: FIG S1 [file mbio.02531-21-sf001.pdf]

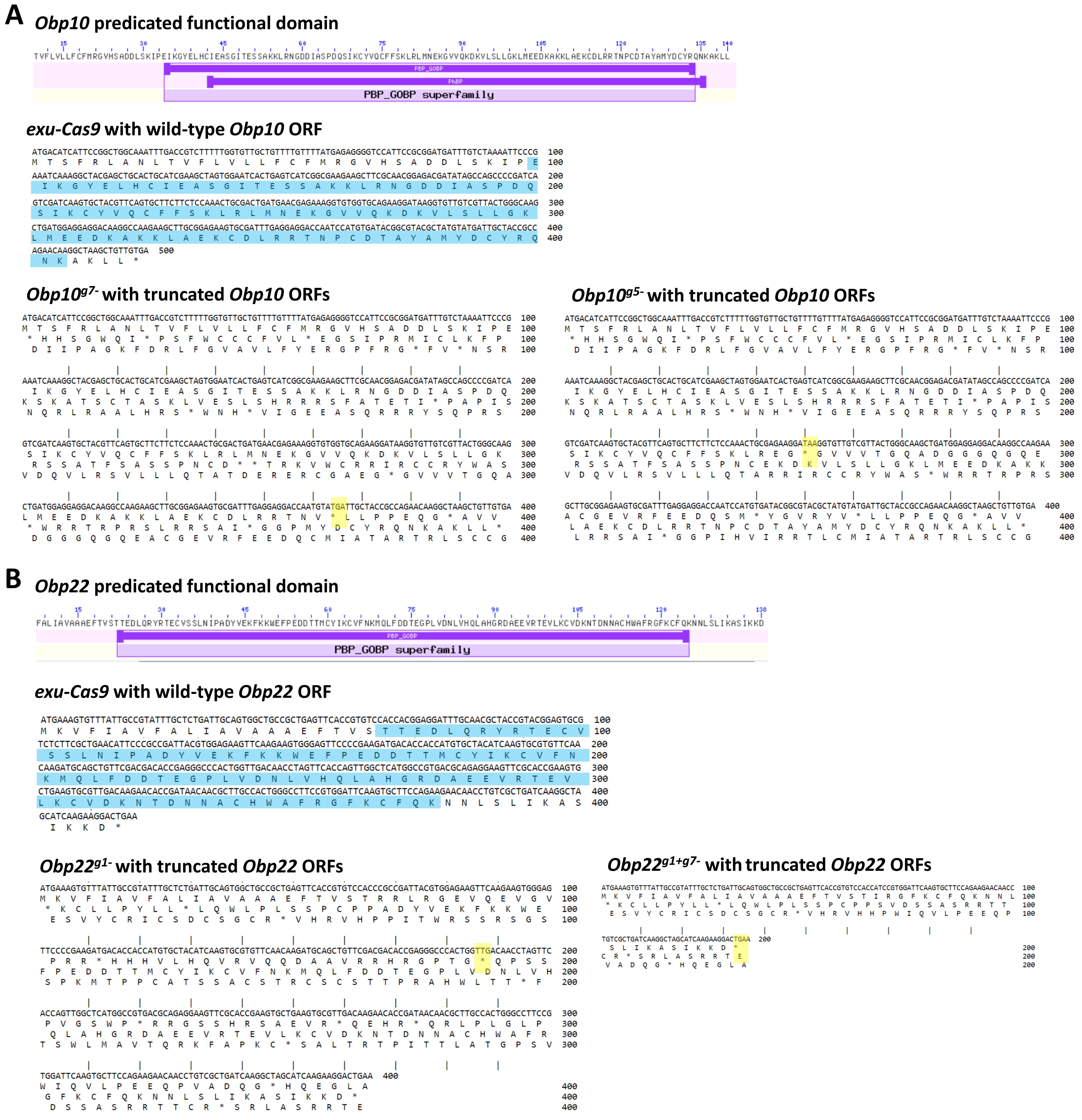
A
Obp10 predicated functional domain
PBP_GOBP
PhBP
PBP_GOBP superfamily
exu-Cas9 with wild-type Obp10 ORF
Obp10g7- with truncated Obp10 ORFs
Obp10g5- with truncated Obp10 ORFs
B
Obp22 predicated functional domain
PBP_GOBP
PBP_GOBP superfamily
exu-Cas9 with wild-type Obp22 ORF
Obp22g1- with truncated Obp22 ORFs
Obp22g1+g7- with truncated Obp22 ORFs

FIG S1
