# Supplementary figures and images for: Pleiotropic Odorant-Binding Proteins Promote Aedes aegypti Reproduction and Flavivirus Transmission
Source: mBio. 2021 Oct 12;12(5):e02531-21. doi: 10.1128/mBio.02531-21 (PMC8510553; doi:10.1128/mBio.02531-21)

**A**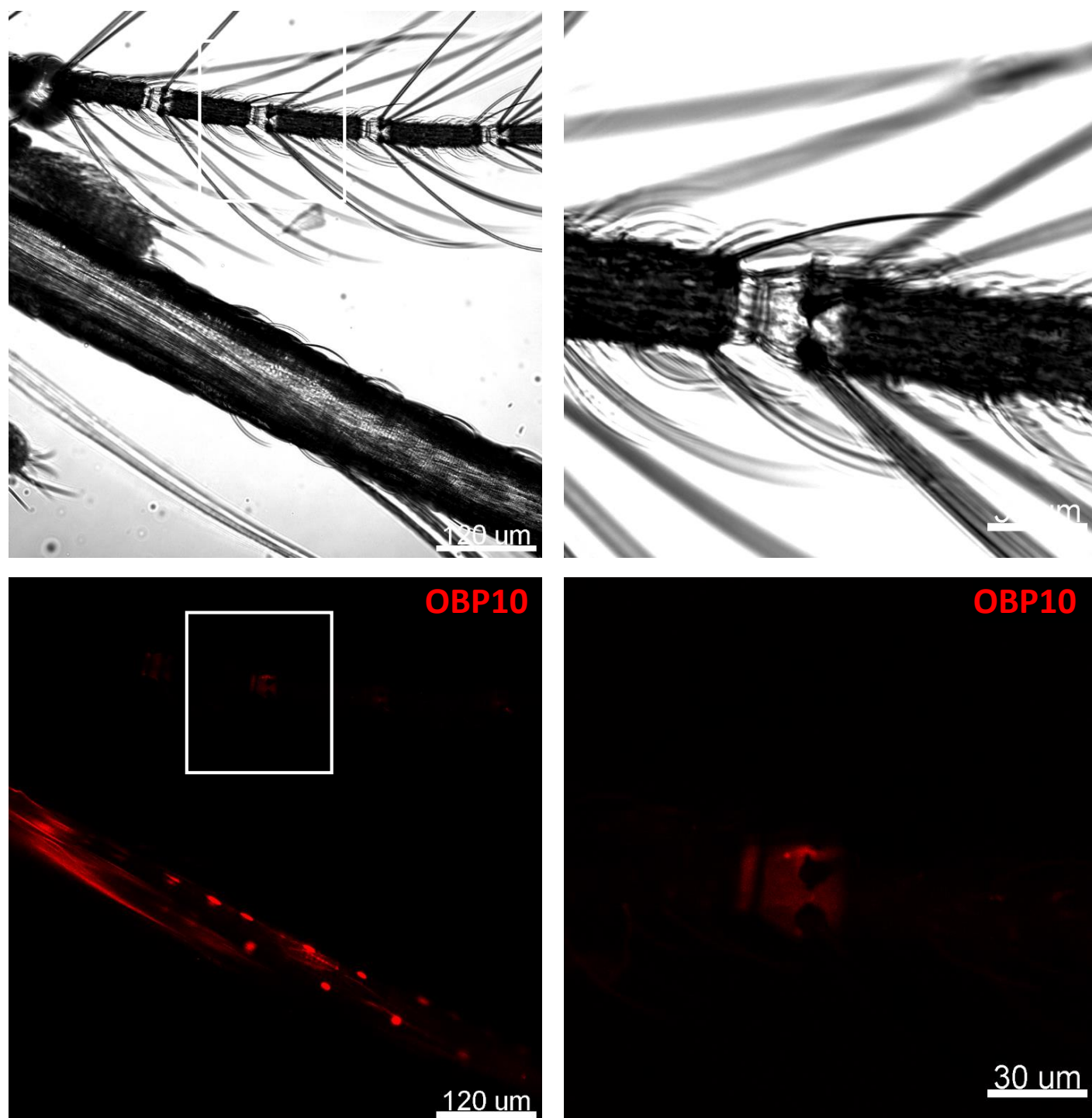**B**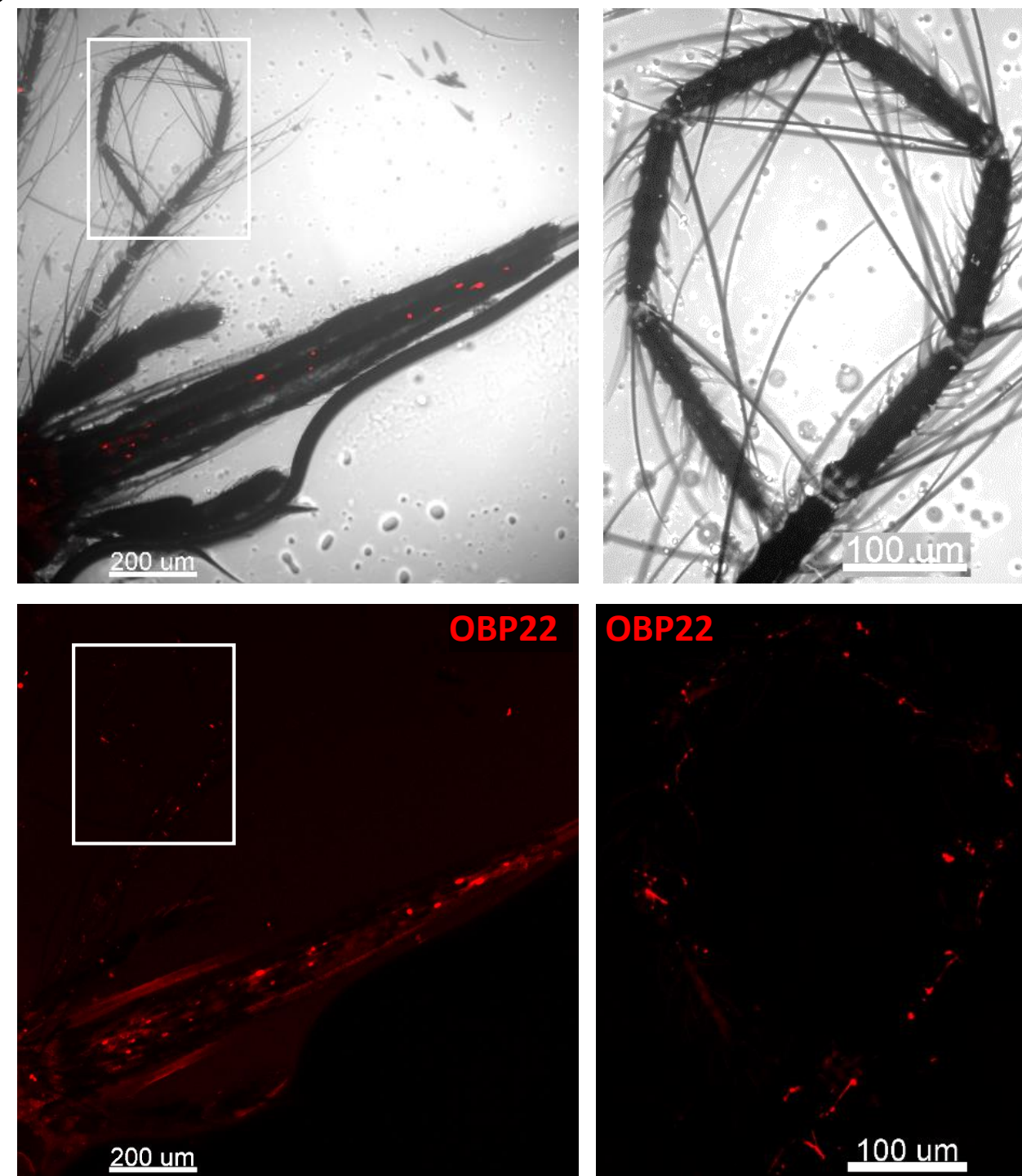**FIG S2**

Supplement: FIG S2 [file mbio.02531-21-sf002.pdf]

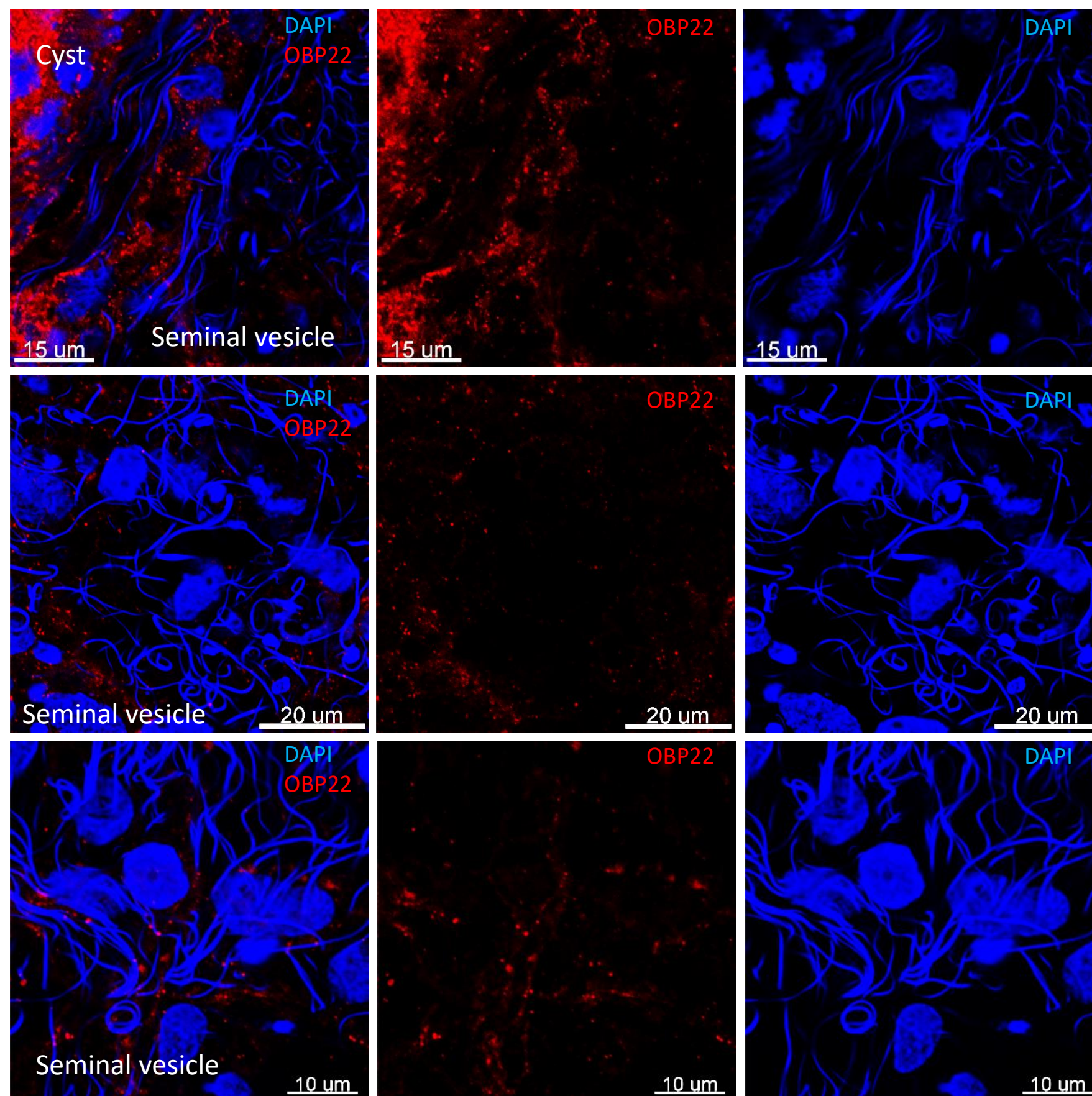

**FIG S3**

Supplement: FIG S3 [file mbio.02531-21-sf003.pdf]

**A**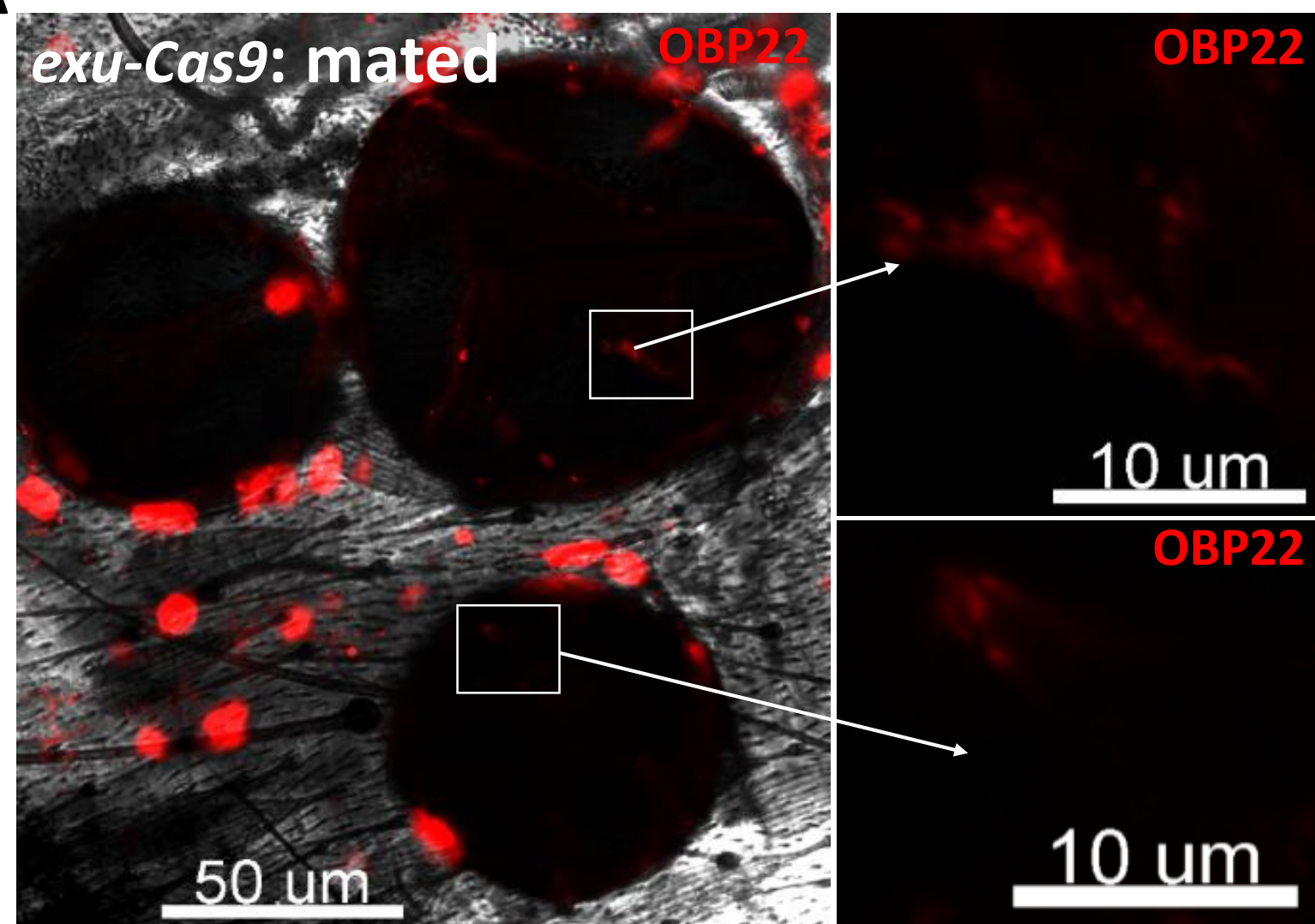**B**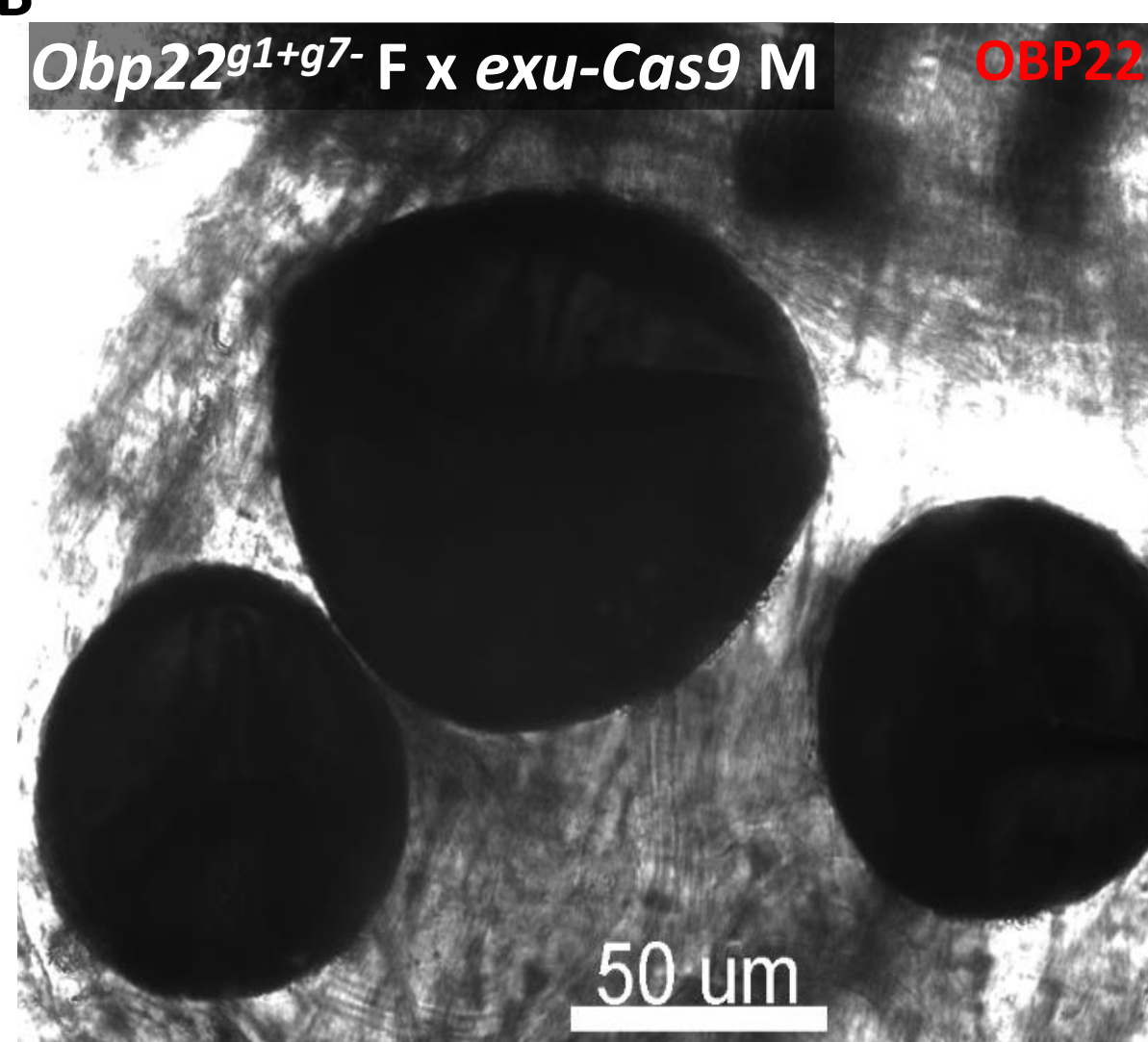

**FIG S4**

Supplement: FIG S4 [file mbio.02531-21-sf004.pdf]

**A**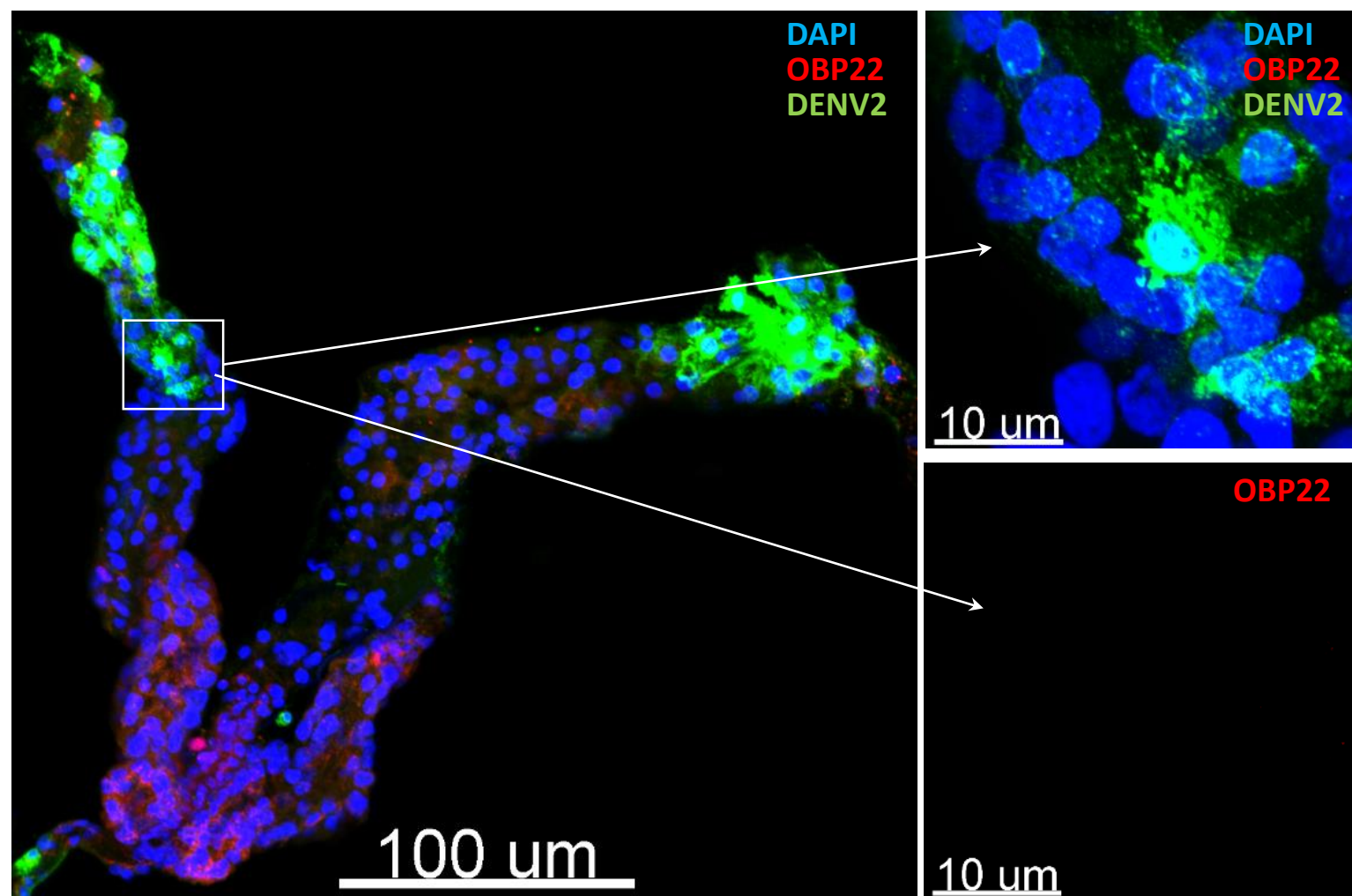**B**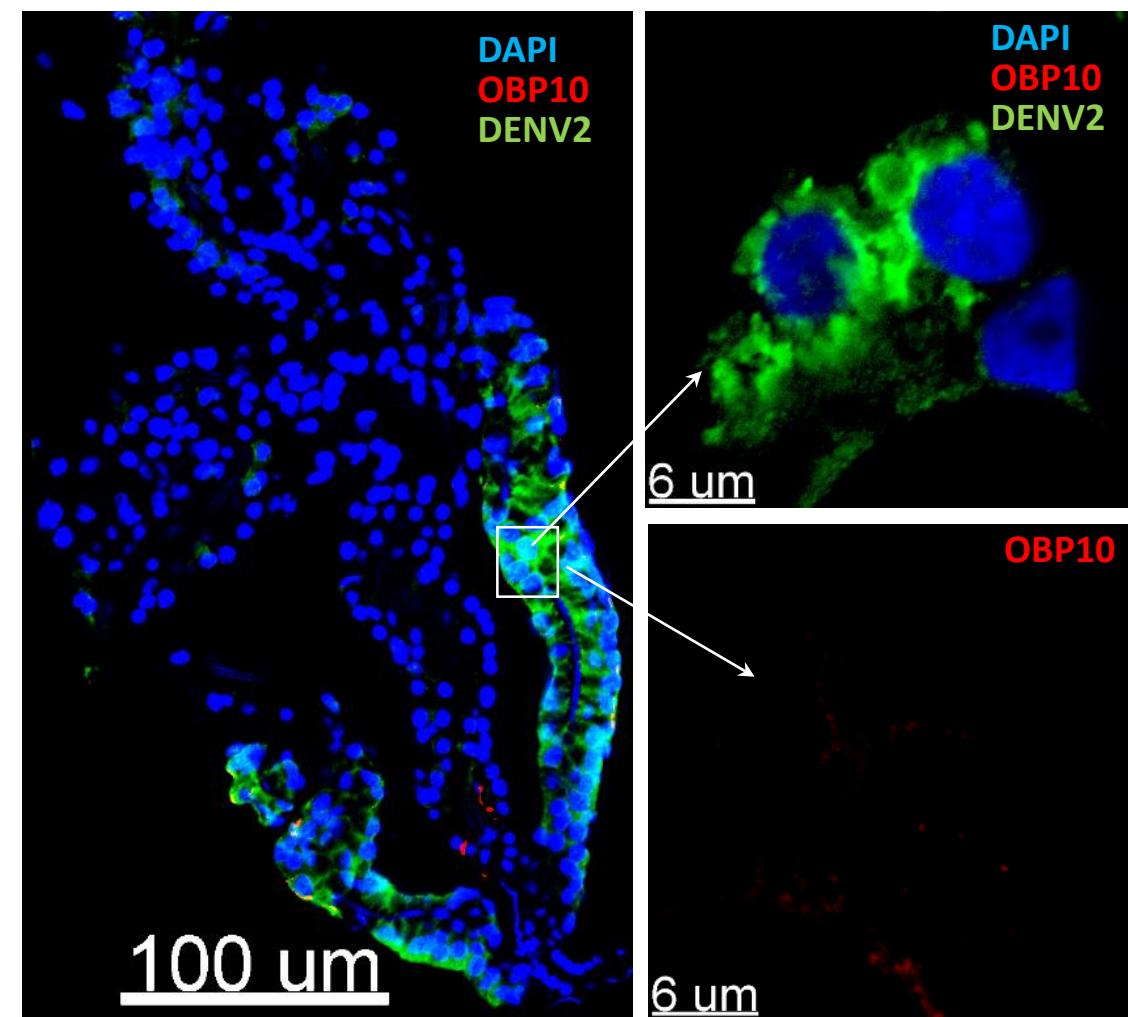**FIG S5**

Supplement: FIG S5 [file mbio.02531-21-sf005.pdf]

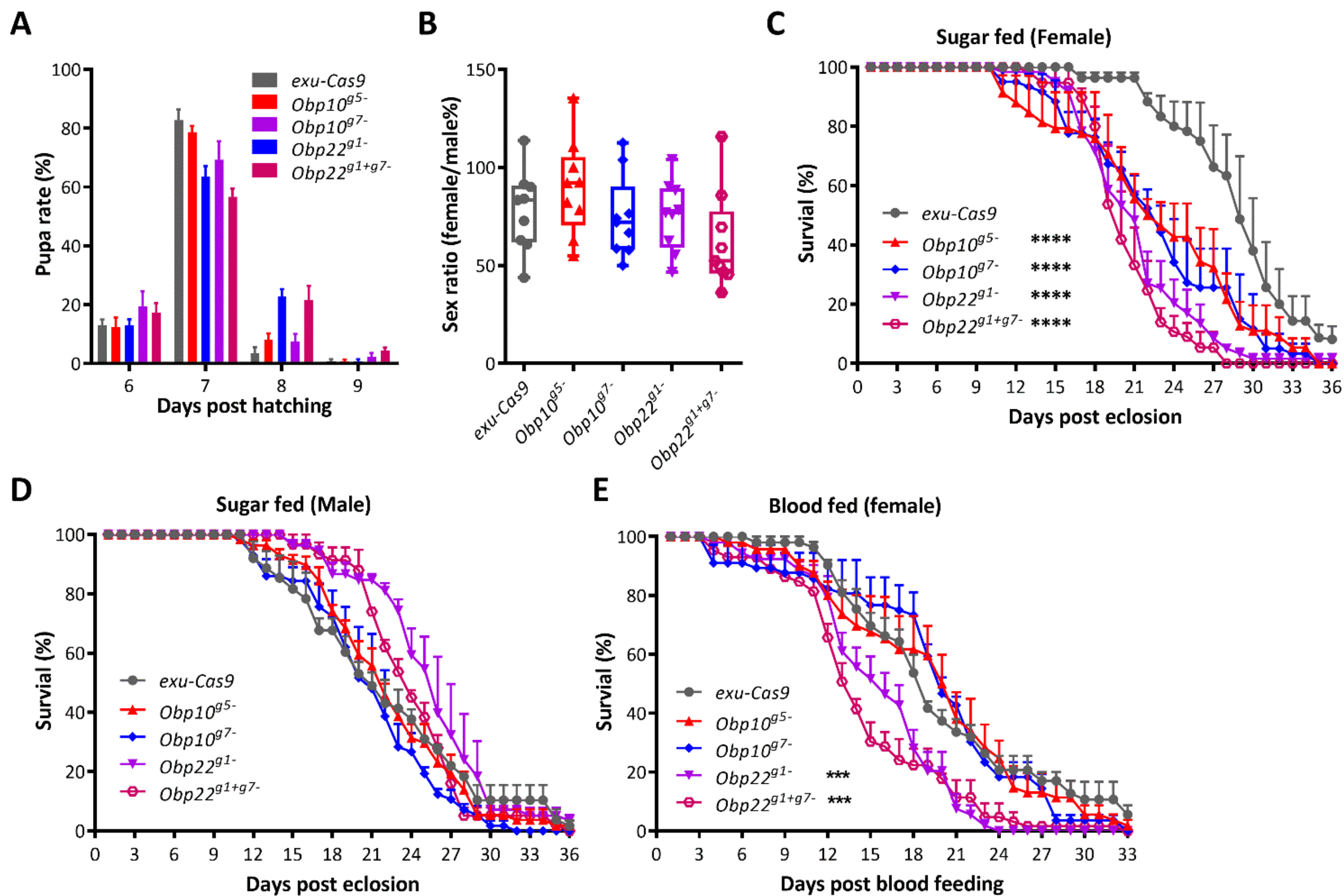

**FIG S6**

Supplement: FIG S6 [file mbio.02531-21-sf006.pdf]
