# Supplementary material for: Pleiotropic Odorant-Binding Proteins Promote Aedes aegypti Reproduction and Flavivirus Transmission
Source: mBio. 2021 Oct 12;12(5):e02531-21. doi: 10.1128/mBio.02531-21 (PMC8510553; doi:10.1128/mBio.02531-21)
Supplement: TABLE S2 [file mbio.02531-21-st002.pdf]

Table S2 List of primers.

Name	Sequence (5' to 3')	Purpose
*Obp10*_sg5-crR-F	TTAATACGACTCACTATAGGCGAGAAAGGTGTGGTGCAGAGTTTTAGAGCTAGAAATAGC	gRNA synthesis
*Obp10*_sg7-crR-F	TTAATACGACTCACTATAGGGTACGCCGTATCACATGGATGTTTTAGAGCTAGAAATAGC	
*Obp10*_sg8-crR-F	TTAATACGACTCACTATAGGCAGCTCGTAGCCTTTGATTTGTTTTAGAGCTAGAAATAGC	
*Obp22*_sg1-crR-F	TTAATACGACTCACTATAGGGTTCACCGTGTCCACCACGGGTTTTAGAGCTAGAAATAGC	
*Obp22*_sg7-crR-F	TTAATACGACTCACTATAGGGCTTGCCACTGGGCCTTCCGGTTTTAGAGCTAGAAATAGC	
sgRNA-R	AAAAGCACCGACTCGGTGCCACTTTTTCAAGTTGATAACGGACTAGCCTTATTTTAACTTGCTATTTCTAGCTCTAAAAC	
Obp10-2F	GTGCTACGTTCAGTGCTTCTTC	PCR
Obp10-2R	GTGATGCTCCGTGCGTTGTAG	
Obp10-8F	GATGAACGAGAAAGGTGTGG	
Obp10-8R	GTACGCCGTATCACATGG	
Obp10-1F	CAGTGTTCGGCATGGAGTTCG	
Obp10-2F	AGAACTTGACCATTTCATGCC	
Obp10-1R	CTCTCGGCTAGAGCTTTCTCG	
Obp10-2R	CTCCTCTTCGTAGGCCCTCTG	
Obp10-Seq-1R	TGCCAGCCGGAATGATGTCA	
Obp10-Seq-1F	GTATGATTGCTACCGCCAG	
Obp10-Seq-2F	CTGTGAGCAGCTGTGTTTAACG	
Obp22-1F	GCCGTATTTGCTCTGATTGCAG	
Obp22-1R	GTAATCGGCGGGAATGTTCAGC	
Obp22-7F	GTGCTGAAGTGCGTTGACAAG	
Obp22-7R	CAGTCCTTCTTGATGCTAGCCTTG	
Obp22-3R	CCAGATGAGATCAAATGCC	
Nix-1F	GATTTTTGTTTTTGTCGTGCAA	
Nix-1R	AATGCAAGTATCATAGGTCAGCA	
Nix-2F	CAAAGAATGATGTGGTTGTCAAA	
Nix-2R	GGGCTTAGTAGCTAAAGATAATTGC	
rpS7-F	ATGGTTTTCGGATCAAAGG	
rpS7-R	CTTGTGTTCAATGGTGGTCTG	
Trypsin202 F	CGA ATG GTA TGT GCC GGT TA	qPCR
Trypsin202 R	CAA CTC CGA CCA GGG TAT TG	
Trypsin712 F	TAA GAA GGT CAA CCG CCA TC	
Trypsin712 R	CGC AGC TAT CGG AGA ATG TTA	
*VgA1*_F	CTCGTTCCCGCTCTGGCAGC	
*VgA1*_R	TGTAGCCGCGACCAATGTCGG	
RT *Obp22* F	ACGCCGAGGAAGTTCGCACC	
RT *Obp22* R	TGCTAGCCTTGATCAGCGACAGG	
RT *rpS7* F	GCAGACCACCATTGAACACA	
RT *rpS7* R	CACGTCCGGTCAGCTTCTTG	
