# Supplementary material for: Pleiotropic Odorant-Binding Proteins Promote Aedes aegypti Reproduction and Flavivirus Transmission
Source: mBio. 2021 Oct 12;12(5):e02531-21. doi: 10.1128/mBio.02531-21 (PMC8510553; doi:10.1128/mBio.02531-21)
Supplement: FIG S7 [file mbio.02531-21-sf007.pdf]

A

AAEL022912
Male determiner gene *Nix* [Source:UniProtKB/Swiss-Prot
AaegL5_152,616,641..152,718,167 (- strand)

AAEL005772
Odorant-binding protein 22, *Obp22*
AaegL5_1:122,385,671..122,386,441 (+ strand)

Apart from *Nix* is around 152,616,641-122,386,441=30,230,200.

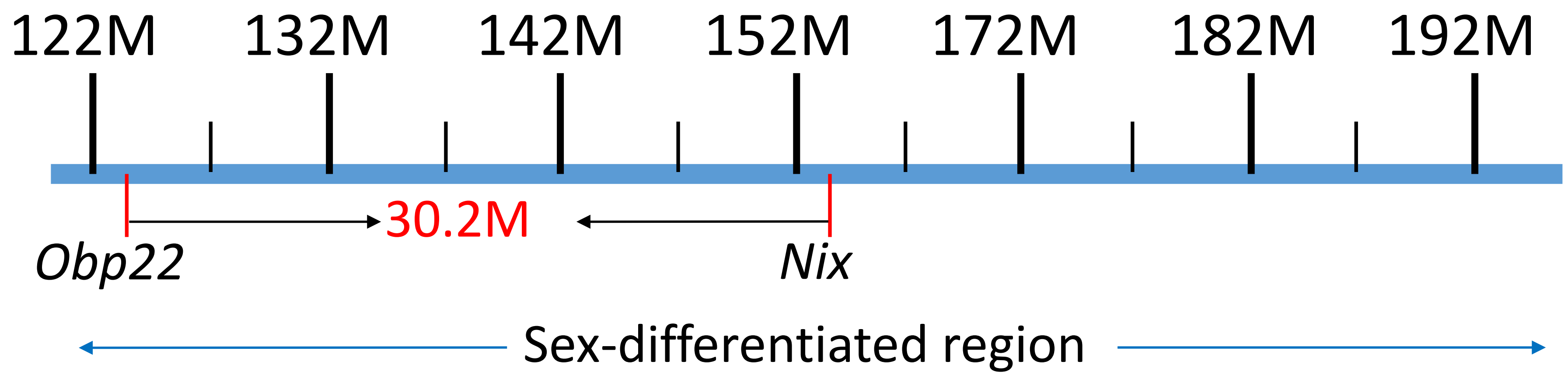

B

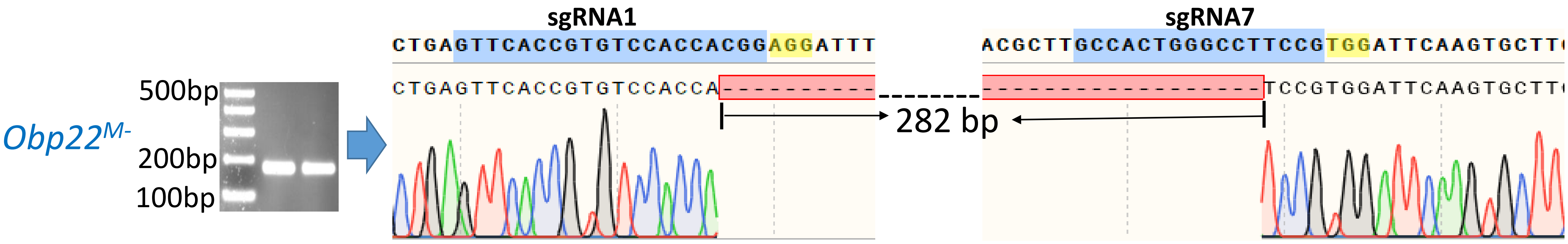

FIG S7
